# Combinatorial targeting of a chromatin complex comprising Dot1L, menin and the tyrosine kinase BAZ1B reveals a new therapeutic vulnerability of endocrine therapy-resistant breast cancer

**DOI:** 10.1186/s13058-022-01547-7

**Published:** 2022-07-18

**Authors:** Annamaria Salvati, Viola Melone, Assunta Sellitto, Francesca Rizzo, Roberta Tarallo, Tuula A. Nyman, Giorgio Giurato, Giovanni Nassa, Alessandro Weisz

**Affiliations:** 1 Laboratory of Molecular Medicine and Genomics, Department of Medicine, Surgery and Dentistry ‘Scuola Medica Salernitana”, University of Salerno, via S. Allende, 1, 84081 Baronissi, SA Italy; 2 Medical Genomics Program, Division of Oncology, AOU ‘S. Giovanni di Dio e Ruggi d’Aragona’, Università di Salerno, 84131 Salerno, Italy; 3 Genome Research Center for Health, 84081 Baronissi, SA Italy; 4 Department of Immunology, Institute of Clinical Medicine, University of Oslo and Rikshospitalet Oslo, 0424 Oslo, Norway

**Keywords:** Breast cancer, Dot1L, Menin, BAZ1B, Estrogen signaling, Endocrine therapy resistance

## Abstract

**Background:**

Targeting vulnerabilities of cancer cells by inhibiting key regulators of cell proliferation or survival represents a promising way to overcome resistance to current therapies. In breast cancer (BC), resistance to endocrine therapy results from constitutively active or aberrant estrogen receptor alpha (ERα) signaling to the genome. Targeting components of the ERα pathway in these tumors represents, therefore, a rational way toward effective new treatments. Interaction proteomics identified several proteins associated with ERα in BC cells, including epigenetic complexes controlling gene transcription comprising the scaffold protein menin and the histone methyltransferase Dot1L.

**Methods:**

We combined chromatin immunoprecipitation, transcriptome sequencing, siRNA-mediated gene knockdown (kd), pharmacological inhibition coupled to cellular and functional assays and interaction proteomics in antiestrogen (AE)-sensitive and AE-resistant human BC cell models to: map menin and Dot1L chromatin localization, search for their common and specific target genes, measure the effects of single or combinatorial knockdown or pharmacological inhibition of these proteins on cell proliferation and survival, and characterize their nuclear interactomes.

**Results:**

Dot1L and menin associate in MCF-7 cells chromatin, where they co-localize in a significant fraction of sites, resulting in co-regulation of genes involved, among others, in estrogen, p53, HIF1α and death receptor signaling, regulation of cell cycle and epithelial-to-mesenchymal transition. Specific inhibitors of the two factors synergize with each other for inhibition of cell proliferation of AE (tamoxifen or fulvestrant)-sensitive and AE-resistant BC cells. Menin and Dot1L interactomes share a sizeable fraction of their nuclear partners, the majority being known BC fitness genes. Interestingly, these include B-WICH and WINAC complexes that share BAZ1B, a bromodomain protein comprising a tyrosine–protein kinase domain playing a central role in chromatin remodeling and transcriptional regulation. BAZ1B kd caused significant inhibition of ERα expression, proliferation and transcriptome changes resulting in inhibition of estrogen, myc, mTOR, PI3K and AKT signaling and metabolic pathways in AE-sensitive and AE-resistant BC cells.

**Conclusions:**

Identification of a functional interplay between ERα, Dot1L, menin and BAZ1B and the significant effects of their co-inhibition on cell proliferation and survival in cell models of endocrine therapy-resistant BC reveal a new therapeutic vulnerability of these aggressive diseases.

**Supplementary Information:**

The online version contains supplementary material available at 10.1186/s13058-022-01547-7.

## Background

The initiation and progression of human cancers, and, in particular breast cancer (BC), lead to multiple genomic imbalances caused, among others, by transcriptional and epigenetic chromatin deregulations [1, 2]. This depends upon the activity of regulatory proteins, such as transcriptional cofactors and chromatin regulators that drive gene signatures defining key functional features of malignant cells, and therefore represent potentially useful targets for innovative and specific therapies. About 70% of newly diagnosed invasive BCs express estrogen receptor alpha (ERα), a ligand-inducible transcription factor, and the presence of this receptor identifies tumors likely to respond to endocrine therapies, based on the use of antiestrogens (AEs) or aromatase inhibitors to block its activity in cancer cells. In ERα positive (ER +) BC, a number functional proteins contribute to the activity of this nuclear receptor by binding to it and regulating gene expression at both transcriptional and post-transcriptional level [3]. Endocrine therapy represents an effective therapeutic strategy against this BC; however, more than 30% of ER + BC are unresponsive to these pharmacological regimens for still unknown reasons [4]. Among the cellular factors able to influence estrogen signaling and effectiveness of endocrine therapy, chromatin regulator enzymes such as epigenetic ‘writers,’ ‘erasers’ and ‘readers’ play a crucial role in the ERα nuclear pathway [5]. To identify molecular targets exploitable to treat ER + tumors resistant to endocrine therapy, in particular AE treatments, we and others focused on the ERα molecular partners comprised in the nuclear multiprotein complexes the receptor requires for its oncogenic activity and involved in epigenetic signaling and chromatin remodeling [6–8]. Among the most promising, the histone lysine N-methyltransferase, H3 lysine-79 specific, Dot1L was found to be a key component of the ERα-mediated transcriptional regulation in BC, where its selective pharmacological inhibition was shown to cause growth arrest and death of AE-sensitive and AE-resistant human ER + BC tumor models [8]. Dot1L functions by catalyzing histone H3 mono-, di- and tri-methylation on lysine-79 and has been linked to several cellular and molecular processes in cancer. In mixed-lineage leukemia, for example, this enzyme associates with MLL fusion proteins, sustaining leukemogenesis via transcriptional regulation mechanisms, and its inhibitors have been clinically tested as therapeutic targets for leukemias carrying this genetic vulnerability [9]. The potential of Dot1L pharmacological blockade for therapy has been demonstrated also in ovarian, prostate and other cancers [8, 10–13]. For solid tumors, Dot1L inhibitors are still far from clinical use, in particular for problems caused by their low tolerability and severe side effects. Combining these with drugs targeting other key factors of cancer cells, in particular other molecular ERα partners in BC, represents a rationale way to try to improve their therapeutic efficacy, allowing, for example, to lower their effective dosage thereby reducing side effects. One such factors is the multiple endocrine neoplasia type 1 gene (MEN1)-encoded protein menin, which has been shown to cooperate with ERα in regulation of the expression of target genes, including ESR1 itself, through chromatin looping mechanisms [14]. Previous studies have demonstrated that menin activates ERα by physically binding to it [15, 16] and can regulate gene expression through the interaction with several epigenetic factors [17–19], including the mixed-lineage leukemia KMT2A-KMT2B protein complex. Inhibition of menin–MLL interaction with small molecules reverses the oncogenic activity of MLL fusion proteins in leukemia [20] or growth of castration-resistant prostate tumors by blocking androgen receptor signaling [21]. Based on the growing body of evidence that targeting the functional interdependence between chromatin remodeling factors and protein complexes they form represents a powerful approach to inactivate essential gene pathways in cancer [1, 2, 22, 23], we investigated the functional relationships between Dot1L and menin in ER + BC cells. Comparing Dot1L and menin effects on BC cell transcriptome and their chromatin binding sites revealed co-recruitment of both proteins in a significant fraction of genome sites, resulting in co-regulation of genes involved in key BC signaling pathways including, among others, estrogen, p53, HIF1α and death receptor signaling, and the control of cell cycle and epithelial-to-mesenchymal transition. Specific Dot1L and menin inhibitors synergized with each other for inhibition of cell proliferation and induction of cell death in AE-sensitive and AE-resistant BC cell lines, accompanied by suppression of ERα expression. Mapping Dot1L and menin BC nuclear interactomes by interaction proteomics demonstrated the presence of a sizable number of shared molecular partners, the majority of which are known BC fitness genes. Interestingly, these include B-WICH and WINAC complexes that share BAZ1B, a bromodomain protein comprising an atypical tyrosine–protein kinase domain, which play a role in chromatin remodeling and transcriptional regulation. BAZ1B silencing caused significant inhibition of cell proliferation and ERα expression, as well as transcriptome changes resulting in inhibition of estrogen, myc, mTOR, PI3K and AKT signaling and metabolic pathways in AE-sensitive and AE-resistant BC cells, revealing a novel therapeutic vulnerability of these cancer cells via inhibition of BAZ1B.

When combined, these results show for the first time a functional interdependence between Dot1L and menin in estrogen-sensitive and AE-resistant breast cancer, and point to BAZ1B as a member of a Dot1L-menin regulatory network exploitable for devising new ways to treat endocrine-resistant BC.

## Methods

### Cell lines

ERα-positive human breast cancer cell line MCF-7 and its tamoxifen-resistant clone MCF-7 tam 1 (ATCC HTB-22 and ATCC-CRL-3435) were purchased from the American Type Culture Collection (ATCC) while ICI-resistant MCF-7/182R-6 cells (16,022,506) were purchased from the European Collection of Authenticated cell cultures. All cell lines used in this study were previously described and characterized by our group [8] and were cultured according to manufacturer’s protocol. Culture medium for tamoxifen- and fulvestrant-resistant cells was supplemented, respectively, with 1 µM 4-hydroxytamoxifen (TAM) or 100 nM fulvestrant (ICI) (Sigma-Aldrich). MCF-7 stably transfected with ERE-TK-LUC plasmid [24] containing the luciferase reporter gene under the control of the estrogen-responsive element was cultured in in Dulbecco's modified Eagle’s medium (Sigma-Aldrich) supplemented with 10% FBS (HyClone, Milan, Italy) and 100 U/ml penicillin, 100 mg/mL streptomycin and 250 ng/mL Amphotericin-B. MCF-7-flag cells were generated by stably expressing full-length-3xFlag-ESR1 (ERα) protein in MCF-7. First, the 3xFLAG-ESR1DNA fragment, amplified from pwPXL vector (kindly provided by Dr. Ruggero [25]), was cloned into pLVX-EF1a-PTS1-IRES-Puromycin (134,665, Addgene) by Clontech In-Fusion HD Cloning Kit. Lentiviral particles containing ERα-encoding RNAs were produced using the Clontech Lenti-X Packaging Single Shots (VSV-G) according to the manufacturer’s protocol and lentivirus particles titer was measured by Lenti-X RT-qPCR Titration Kit (Takara, Clontech). Subsequently, MCF-7 cell lines were infected lentiviral particles and positive selection was performed growing cells in medium complemented with 0,5 μg/ml of Puromycin (for 5–6 days). MCF-7-flag were cultured in in Dulbecco’s modified Eagle’s medium (Sigma-Aldrich) supplemented with 10% FBS (HyClone, Milan, Italy) and 100 U/ml penicillin, 100 mg/mL streptomycin and 250 ng/mL Amphotericin-B.

### Antibodies and compounds

The antibodies used for co-immunoprecipitation and/or western blot analyses were the following: anti-ERα C-terminal (F-10 sc-8002, Santa Cruz Biotechnology, Dallas, Texas), anti-ERα (ab3575) and anti-KMT4/Dot1L (ab72454) from Abcam (Cambridge, UK), anti-Dot1L (A300-953A), antimenin (A300–105 A) and anti-WSTF (BAZ1B; A300-446A) from Bethyl Laboratories (Montgomery, Alabama), anti-β-actin (A1978) from Sigma-Aldrich (Milan, Italy), Rabbit IgG Isotype Control (31,235) from Thermo Fisher (Milan, Italy). Compounds used for cell treatments were 4-hydroxytamoxifen (TAM; H7904, Sigma-Aldrich), fulvestrant (ICI; I4409, Sigma-Aldrich), 17β-estradiol (E887-5G, Sigma-Aldrich). Pharmacological inhibition was performed with EPZ004777 (S7353; only for RNA-seq experiments) and Pinometostat EPZ5676 (S7062; for all the other experiments included in the manuscript) for Dot1L and with MI-136 (S7815) and MI-503 (S7817) for menin, all purchased from Selleckchem (Houston, TX, USA). All inhibitors were dissolved in DMSO (Sigma-Aldrich), which was therefore used as a vehicle.

### Chromatin immunoprecipitation

For each experimental setting, MCF-7 cells were washed and subjected to cross-linking in 0.75% formaldehyde for 10 min at room temperature. Formaldehyde was quenched by the addition of 125 mM glycine for 8 min. Cells were washed and chromatin extraction was performed as previously described [26]. Chromatin immunoprecipitation (ChIP) assays were performed incubating isolated chromatin with 100 μl Dynabeads M-280 Sheep Anti-Rabbit IgG (Thermo Fisher), previously equilibrated overnight at 4 °C with 10 μg of antimenin antibody, anti-Dot1L or Control antibodies. Bead washing was performed as previously described [8]. Chromatin-associated proteins were eluted by the addition of Laemmli buffer and resolved by SDS-PAGE and western blotting.

### ChIP-seq data analysis

ChIP-Seq data of menin were downloaded from GEODataset (GSE85317) and for Dot1L were obtained from Nassa et al. [8]. Data analysis was performed as described by Tarallo et al. [27]. In detail, the raw sequence files generated (.fastq) underwent quality control analysis using FASTQC (http://www.bioinformatics.babraham.ac.uk/projects/fastqc/). Reads were aligned to the reference human genome assembly (hg38) using bowtie [28], allowing up to one mismatch and considering uniquely mappable reads. Duplicated reads were removed using Picard tools v 2.9.0 (MarkDuplicates; https://broadinstitute.github.io/picard). For each biological replicate, peak calling was performed using MACS2 [29] with *p*-value set to 0.05. The peaks obtained for each biological replicate were combined using MuSERA [30]. The annotation of peaks to the nearest gene was performed combining the information obtained using the annotatePeaks.pl function of HOMER [31] and the Annotation and Statistics of Genomatix Software suite. Comparison, integration and quantification were performed using seqMINER [32]. Overrepresented sequence motifs for known transcription factors, according to motif descriptors in the JASPAR database, were determined using PScan-ChIP [33]. Only overrepresented motifs with *p* value ≤ 1E-10 were considered.

### Co-immunoprecipitation, mass spectrometry and data analysis

For Dot1L, menin and BAZ1B co-immunoprecipitation, anti-Dot1L, antimennin, anti-BAZ1B or anti-IgG Isotype Control were conjugated overnight at 4 °C with Dynabeads M-280 Sheep AntiRabbit IgG (Thermo Fisher Scientific). Day after, nuclear protein extract from MCF-7 cells in normal growing condition was prepared as previously described [34] and incubated with conjugated beads/antibodies at 4 °C for 2 h/overnight. IPs were subsequently washed with IPP150 buffer (7.14 mM HEPES pH 7.5, 8.92% glycerol, 150 mM NaCl, 0.54 mM MgCl2, 0.07 mM EDTA pH 8 and 1 × protease inhibitors) and Wash Buffer (50 mM Tris–HCl pH 7.6, 150 mM NaCl and 1 × protease inhibitors) and suspended either in Laemmli buffer for western blot analysis or in 100 μl of 100 mM ammonium bicarbonate before tryptic digestion, LCMS/MS and data analysis. For mass spectrometry (MS) analyses, IPs from three biological replicates of samples (Dot1L or menin) and controls (IgGs) were analyzed. Protein digestion was performed by on-beads digestion with trypsin (Promega). The resulting peptides were desalted and concentrated before MSby the STAGE-TIP method using a C18 resin disk (3 M Empore). The peptides were eluted with 0.1% TFA/50% ACN, dried and solubilized in 7 μL 0.1% FA for MSanalysis. Each peptide mixture was analyzed on an Easy nLC1000 nano-LC system connected to a quadrupole Orbitrap mass spectrometer (QExactive HF, ThermoElectron, Bremen, Germany) equipped with a nanoelectrospray ion source (EasySpray/Thermo) using a 60 min separation gradient. The resulting MS raw files of controls, Dot1L and menin samples were submitted to the MaxQuant software (version 1.6.1.0) for protein identification and quantitation using the Andromeda search engine. MaxQuant search was done against the UniProt Human database (September 2018). Carbamidomethyl (C) was set as a fixed modification and protein N-acetylation and methionine oxidation were set as variable modifications. First search peptide tolerance of 20 ppm and main search error 4.5 ppm were used. Trypsin without proline restriction enzyme option was used, with two allowed miscleavages. The minimal unique + razor peptides number was set to 1, and the allowed FDR was 0.01 (1%) for peptide and protein identification. To obtain the list of Dot1L and menin molecular partners, we compared each dataset to the control (IgG). We considered as Dot1L or menin associated proteins those molecules identified only in samples (at least in 2 out of 3 sample biological replicates and absent in the controls) or those supported by statistical analysis performed as follows: a permutation (10,000)-based T-test applied to MaxQuant protein ‘Intensities’ values (FDR ≤ 0.05) between samples (Dot1L and menin) and controls (IgG) and a fold change cutoff ≥ I quartile.

#### UALCAN

The comprehensive web resource UALCAN (http://ualcan.path.uab.edu) [35] was used to carry out analysis based on RNA sequence and clinical data relative to 31 cancer types from The Cancer Genome Atlas (TCGA) and Clinical Proteomic Tumor Analysis Consortium (CPTAC) databases. Data from 566 luminal-like subtype breast cancers and 114 normal breast tissue samples were collected and DOT1L, MEN1 and BAZ1B mRNA expression levels were compared between cancer and normal tissues.

### BAZ1B knockdown

Functional assays and RNA sequencing experiment were performed after BAZ1B knockdown. MCF-7 cells were seeded into 96-well plates (3 × 10^4^ cells/well) in standard growth medium and transiently transfected by using Lipofectamine RNAiMax (13,778–150, Invitrogen) diluted in Optimem medium, according to the manufacturer's instructions. 0.5 pM/well of two validated BAZ1B siRNAs (s17208, s17210) or scramble Silencer Select Negative Control (SiSel_NC1) (Ambion, Thermo Fisher Scientific) were added in quadrupled/sixfold each one and incubated for 72 h at 37 °C/5% CO_2_.

### Cell proliferation assay

Cell proliferation assays were performed using the MTT assay (M6494, Thermo Fisher Scientific). Briefly, cells were seeded sixfold into 96-well plates at a density of 1.4–3 × 10^4^ cells/well and treated with increasing concentrations of EPZ5676, MI 136, MI 503 or indicated combinations of drugs and siRNA (DOT1L: s39010; MEN1: s8682). DMSO, 100 nM TAM, 100 nM ICI or scramble were used as control. After incubation, 72 h for knockdown condition and 3, 6 and 9 days for dose–response assay, cell proliferation was evaluated by adding 1 mg/mL of 3-(4,5-dimethylthiazol-2-yl)-2,5-diphenyltetrazolium bromide (MTT) (M6494, Thermo Fisher) for well and then the plates were returned to the incubator for 4 h. After solubilization of formazan crystals, absorbance was measured at 570 and 620 nm (background) wavelengths by the VICTOR Multilabel Plate Reader (PerkinElmer, Milan, Italy).

### Caspase 3/7 assay

Apoptosis was assayed with fluorimetric detection of caspase-3/7 activity, according to the instructions of Amplite™ Fluorimetric Caspase 3/7 Assay Kit (AAT Bioquest, Inc). In cells, seeded, as previously described, in Clear Bottom Plate (CC3603, Corning) 100 µL/well/96-well plate of Caspase 3/7 working solution were added and incubated at room temperature for 1 h. The fluorescence intensity was measured at Ex/Em = 490/525 nm (Cutoff = 515 nm).

### Luciferase assays and western blot assay

For luciferase reporter gene assays, cells were plated in 96-well plates at a density of 3 × 10^4^ cells/well in the above described experimental setting. After 72 h, cells were harvested in lysis buffer, and luciferase activity was measured using the Luciferase Assay Reagent (Promega Corp.) according to the manufacturer's instructions. The values obtained were normalized to the protein concentrations measured using the Bradford assay. For each condition, average luciferase activity was calculated from the data obtained from four independent replicates. The cell lysate was also suspended in Laemmli buffer and analyzed by western blotting, according to standard protocols. Briefly, protein samples extracts were denatured, separated on 7% or 10% polyacrylamide and 0.1% SDS (SDS-PAGE) and transferred onto a nitrocellulose blotting membrane (GE Healthcare, Milan, Italy). Following blocking with 5% nonfat dry milk in TBST buffer (0.01 m Tris–HCl, pH 8.0, 0.15 m NaCl and 0.1% Tween 20), membranes were immunoblotted overnight with different primary antibodies. After extensively washing with TBST, the primary Abs were detected by horseradish peroxidase-conjugated secondary Abs (GE Healthcare) and revealed by chemiluminescence and autoradiography. Densitometry was performed by ImageJ software analysis [36].

### RNA extraction and sequencing

Total RNA was extracted using TRIzol (Thermo Fisher), according to the manufacturer’s instructions. In brief, 0.3–1 mL of TRIzol™ Reagent for 3 × 10^4^ cells was added directly to the culture dish to lyse the cells by pipetting several times. Before use, RNA concentration was determined by using Quant-IT RNA Assay Kit-High Sensitivity and a Qubit Fluorometer (Life Technologies, Monza, Italy) and its quality and integrity assessed with the Agilent 4200 Tapestation System (Agilent Technologies, Milan, Italy). For RT-qPCR, cell lines were treated with increasing concentrations of EPZ5676, MI 136, MI 503 or combinations of drugs and siRNAs, as described in the results section. For RNA sequencing, cell lines transfected with each BAZ1B siRNAs were prepared from 6 miniwells and pooled. A nontarget siRNA (scramble) was used as negative control. Indexed libraries were prepared starting from 150 ng total RNA according to Illumina Stranded Total RNA prep Ligation with Ribo-Zero Plus kit (Illumina Inc., San Diego, CA, USA). Final libraries were sequenced at a concentration of 0,6 pM/lane on the Novaseq 6000s4 v 1.5 platform (Illumina Inc.). The raw sequence files generated (.fastq files) underwent quality control analysis using FASTQC (http://www.bioinformatics.babraham.ac.uk/projects/fastqc/) and adapter sequences were removed using Trimmomatic version 0.38 [37]. Filtered reads were aligned on human genome (assembly hg38) considering genes present in GenCode Release 36 (GRCh38.p12) using STAR v2.7.5a with standard parameters [38]. Quantification of expressed genes was performed using featureCounts [39] and differentially expressed genes were identified using DESeq2 [40]. A given RNA was considered expressed when detected by at least ≥ 10 raw reads. Differential expression was reported as |fold change| (FC) ≥ 1.5 along with associated adjusted *p*-value or *p*-value ≤ 0.05 computed according to Benjamini-Hochberg. RNA-Seq data from MCF-7 cells subjected to MEN1 pharmacological blockade was downloaded from GEODataset (GSE86316) [14]. RNA-seq data for DOT1L pharmacological blockade with EPZ004777 are from Nassa et al. [14]. The analysis has been performed as described above. Functional analysis of differentially expressed transcripts has been performed using IPA (Ingenuity Pathway Analysis, QIAGEN) and Gene Set Enrichment Analysis (GSEA) [41].

### RT-qPCR

cDNA was synthesized starting from 1 μg total RNA for each sample using Random Hexamer (Tetro cDNA Synthesis Kit, Bioline, Memphis, Tennessee). RTqPCRs were performed in triplicate on a Lightcycler 480 instrument (Roche) using SensiFAST SYBR Lo-ROX kit (Bioline), according to the manufacturer’s instructions. All values were normalized to RPLP0 mRNA. The primers used for qPCR are:

**Table:** 

for BAZ1B:	*Forward primer*: GGGCTCAGACACAGATGACA
	*Reverse primer*: TGGGGCTCAAACTTCACAAT
for DOT1L:	*Forward primer*: CCAGACTGACCAACTCGCACAC
	*Reverse primer*: AGAAATCCTAGTTACCTCCAACTGT
for ESR1:	*Forward primer*: ACCCTCCATGATCAGGTCCA
	*Reverse primer*: CTGGTTCCTGTCCAAGAGCA
for MEN1:	*Forward primer*: GGAGCTGGCTGTACCTGAAA
	*Reverse primer*: GCAATGCCCTTGTGGTAGAG
for RPLPO:	*Forward primer*: CCATCAGCACCACAGCTTC
	*Reverse primer*: GGCGACCTGGAAGTCCAACT
for TFF1:	*Forward primer*: GTGGTTTTCCTGGTGTCACG
	*Reverse primer*: TCACATCCTCTTCTGGAGGG

### Drug combination analysis

The additive and synergistic effects were determined by combining increasing concentration of EPZ5676, MI 136 and MI-503 after 9 days of treatment in sensitive and resistant cell lines. The data were analyzed with the freely available Combenefit software [42], determining synergy/antagonism by Loewe model.

### Statistical analysis

Statistical analyses were performed using *R* (version 4.0.2). Error bars represent means ± SD. Statistical differences were determined using an unpaired two-sample t test. Values of *p* ≤ 0.05 were considered to be statistically significant. Concerning Chi-Seq, RNA-Seq and MS analysis, specific statistical parameters used are detailed in the corresponding Methods sections.

## Results

### Dot1L and menin are co-recruited in BC cell chromatin

**Fig. 1:**
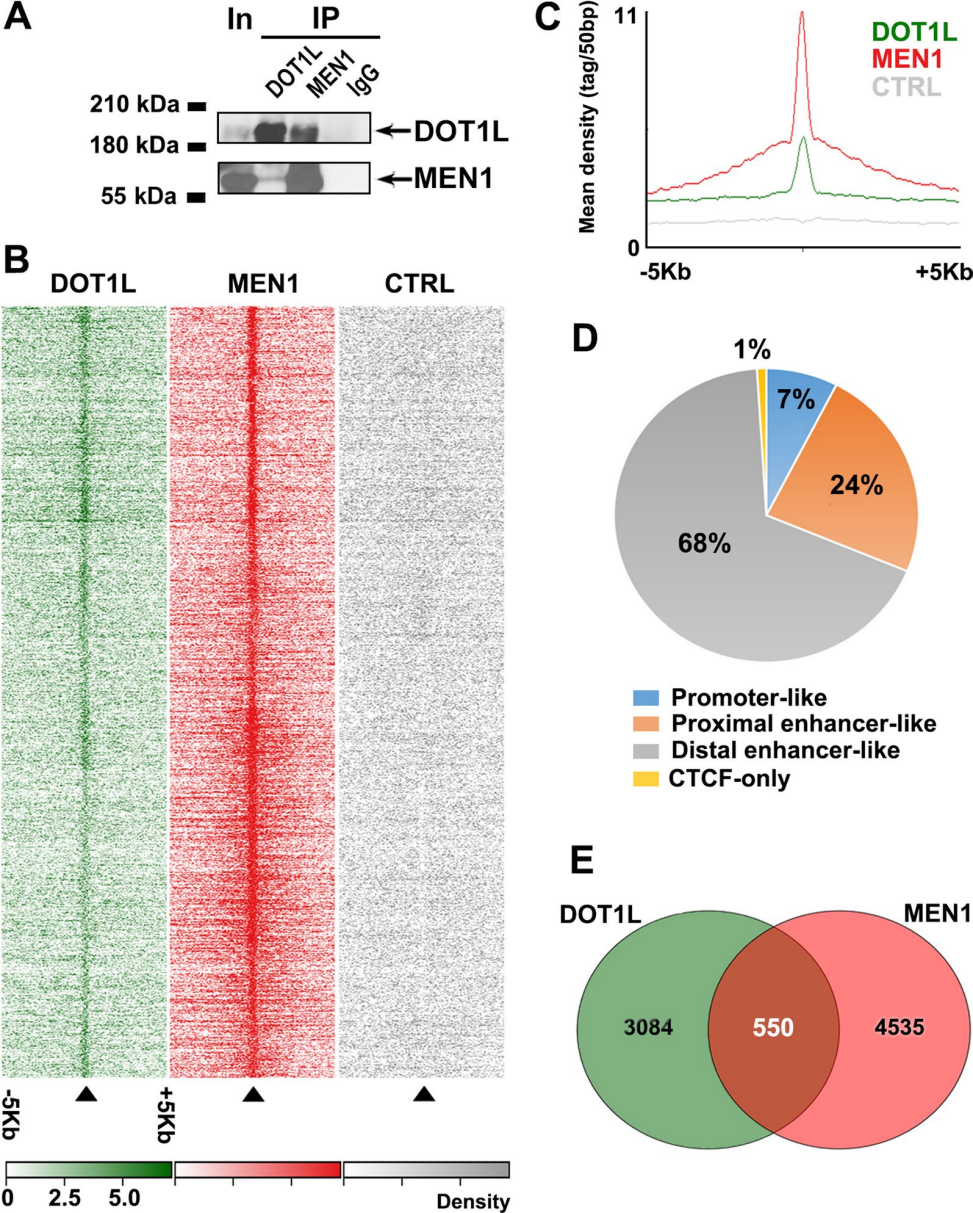
Dot1L and menin binding to BC cells chromatin. **A** ChIP–western blot showing Dot1L and MEN1 co-recruitment on chromatin. IgG was used as negative control. **B** Heatmap showing read density within 10 kb regions centered on Dot1L and menin binding sites in MCF-7 cells. Control (CTRL) was obtained using nonspecific Abs. **C** Mean read density within and around Dot1L and menin co-localized binding sites. **D** Pie chart showing the distribution of Dot1L + menin shared binding sites within the genome. **E** Venn diagram showing the number of expressed transcripts harboring specific and common Dot1L and menin binding sites

Although Dot1L and menin have both been found to be functional partners of ERα in BC cells, where they can physically associate with the receptor on chromatin, the possibility of an interplay between these two factors on transcriptional regulation by ER has not been investigated yet. This possibility is supported by the finding that the genes encoding these proteins (DOT1L and MEN1, respectively) are expressed at significantly higher levels in the majority of luminal-like BCs, compared to normal mammary gland (Additional file 1: Fig. S1A) and, more interestingly, can be often found co-expressed in the same ERα + tumors (Additional file 1: Fig. S1B). When combined with the known activity of each of these two ER binding factors on regulation of gene transcription and epigenetic control of chromatin activity, these evidences led us to further investigate the possibility of a physical/functional cooperation between these two proteins in ERα + BC cell nuclei. Indeed, as shown in Fig. 1A, chromatin IPP coupled to western blotting (ChIP-WB) showed that they are found associated in MCF-7 cell chromatin. Aligning their respective cistromes, mapped under the same conditions and comprising 14,093 binding sites for Dot1L [8] and 8,994 sites for menin [14], we identified 700 binding sites where the two proteins co-localize in chromatin (Fig. 1B, C). Interestingly, the majority of these Dot1L + menin binding sites map to proximal or distal enhancer-like elements, with a fraction surrounding promoter regions, as expected for transcription regulatory complexes (Fig. 1D). When considering expressed genes harboring Dot1L and menin binding sites, about 14% of their transcription units bind both factors (Fig. 1E). Interestingly, half of Dot1L + menin binding sites are shared also by ERα (Additional file 1: Fig. S2), supporting our initial hypothesis that the two factors can indeed play, in combination, an active role in transcriptional regulation of specific gene sets by this receptor. A search for overrepresented transcription factor binding motifs within the shared binding regions revealed a prevalence of ERE (estrogen response element) or ERE-like sequences in ERα + Dot1L + menin (Additional file 1: Fig. S2) sites, suggesting that ER binding could drive interaction of the other two factors on specific chromatin locations. On the other hand, CDE, NRF1, ZF15 and ZF36, binding matrices result particularly enriched in Dot1L + menin binding sites (Additional file 1: Fig. S2). CDE (Cell cycle Dependent Element) has been shown to control cell cycle gene transcription in G2 and mitosis [43], the nuclear respiratory factor-1 (NRF-1) in known to act in concert with estrogen and its receptors to regulate nuclear-encoded mitochondrial genes [44] and ZF15 and ZF36, members of C2H2 zinc finger transcription factor family, are recruiters of chromatin modifiers to the genome [45]. When combined, these data suggest that, in BC cells, Dot1L and menin, via different transcription factors including also ERα, associate together to genomic regions such as enhancers and promoters where they could cooperate to regulate gene pathways.

### *Pharmacological inhibition of Dot1L and menin interferes with estrogen signaling and causes growth arrest and death of ER* + *AE-resistant BC cells*

**Fig. 2:**
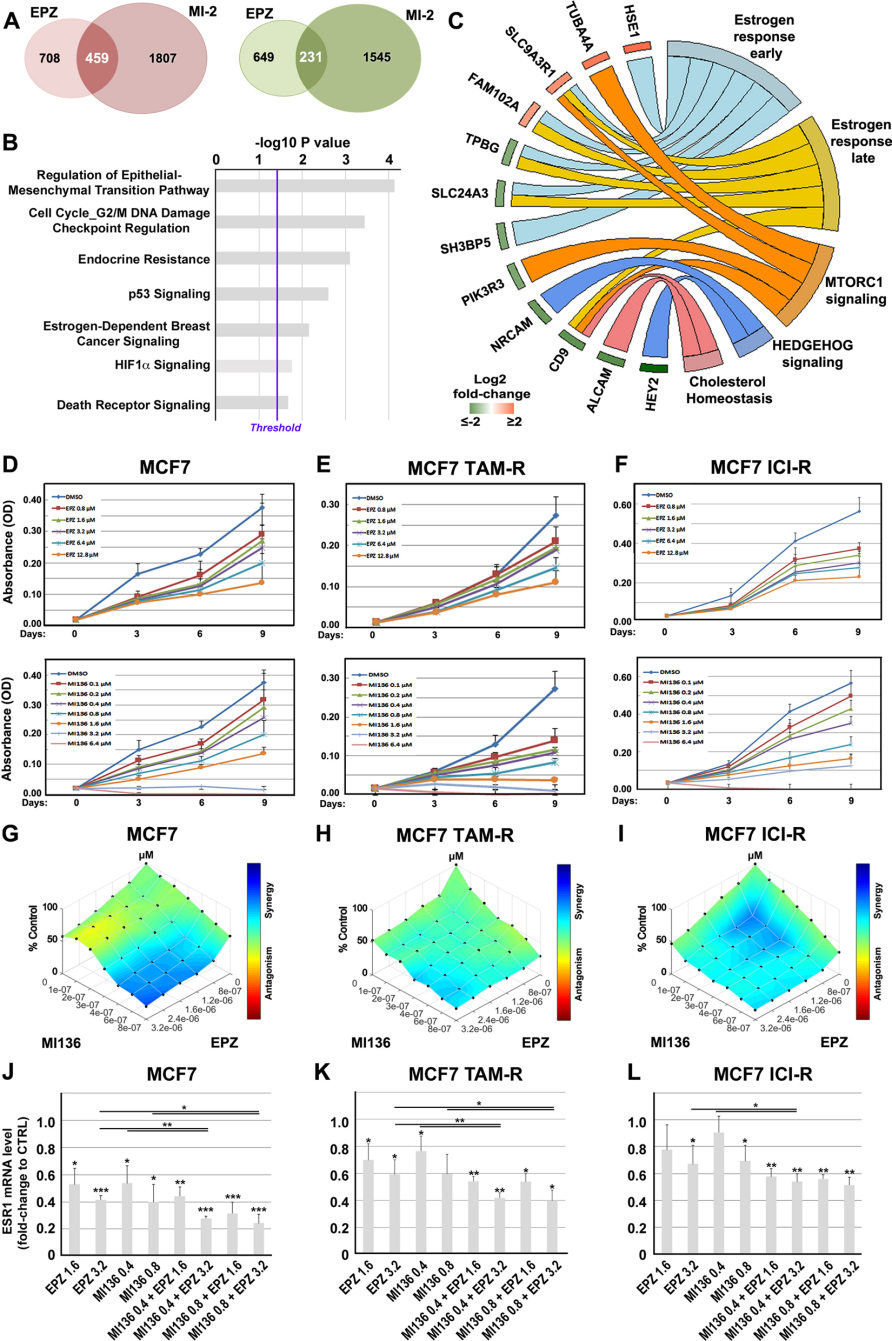
Impact of Dot1L and menin blockade on gene expression and cell functions in BC cells. **A** Venn diagram showing the number of specific and common transcripts up-regulated (red) and down-regulated (green) following EPZ or MI-2 treatment. **B** Bar charts from KEGG functional enrichment analysis showing statistically significant common pathways deregulated upon MCF-7 cell treatment with EPZ and MI-2. **C** Circos plot showing common up-regulated (red) and down-regulated (green) transcripts following EPZ and MI-2 treatment harboring Dot1L + menin binding sites and the statistically significant (*p* ≤ 0.05) pathways they are involved in. Effect of Dot1L and menin pharmacological blockade on AE-sensitive (MCF7; **D**) tamoxifen (MCF7 TAM-R; **E**)- or fulvestrant/ICI (MCF7 ICI-R; **F**)-resistant BC cells following increasing concentrations of EPZ (up) or MI-136 (bottom) after 3, 6 and 9 days. DMSO was used as control. Data are presented as mean ± SD from six independent replicates. Combenefit software was used to generate dose–response surface curves in AE-sensitive (MCF7; **G**) tamoxifen (MCF7 TAM-R; **H**)- or fulvestrant/ICI (MCF7 ICI-R; **I**)-resistant BC cells, according to D-R Lowe model, combining increasing dose of EPZ and MI-136 for 9 days. Color scale bar indicate the level of synergy (blue) or antagonism (red) at each combination. Reverse transcription quantitative real-time polymerase chain reaction (RT-qPCR) analysis of ESR1 () mRNA level in AE-sensitive (MCF7; **J**) tamoxifen (MCF7 TAM-R; **K**)- or fulvestrant/ICI (MCF7 ICI-R; **L**)-resistant BC cells following EPZ or MI-136 as single agents and in combination after 9 days of treatment. RT-qPCR results shown are the mean ± SD of triplicate determinations from a representative experiment. Asterisks indicate statistically significant differences (*p ≤ 0.05, **p ≤ 0.01, ***p ≤ 0.005) to CTRL or to single treatment (black bars)

To investigate the functional significance of Dot1L-menin association to BC cell genome, we compared transcriptome data generated upon pharmacological inhibition of Dot1L with EPZ [8] with the same observed following menin blockade by MI-2 [14]. EPZ and MI-2 caused significant changes in expression of a common set of 459 up- and 231 down-regulated transcripts (Fig. 2A and Additional file 2: Table S1), including several transcripts shown to be regulated directly by ERα in BC cells [46]. This results in activation of p53 and death receptor signaling and, as expected, inhibition of estrogen-dependent signaling (Fig. 2B). Each of the two drugs alone elicited specific gene expression changes that impacted distinct signaling pathways. Of note, EPZ mainly affected chromosomal replication linked to cell cycle, the cytoskeleton regulator stathmin and the PD-1 and PD-L1 immune checkpoint pathway, PPAR signaling and DNA methylation-mediated transcriptional repression (Additional file 1: Fig. S3A). On the other hand, MI-2 targeted EIF2, IL-2, IL7 and VEGF signaling and the cholesterol biosynthesis genetic pathway (Additional file 1: Fig. S3B). Interestingly, 38 transcription units encoding mRNAs affected by both EPZ and MI-2 contain Dot1L + menin binding sites. These comprise, among others, those belonging to early and late estrogen response (Fig. 2C). The observation that the functional interplay between Dot1L and menin has an active part in the mitogenic and cell survival effects of estrogen-sensitive and AE-resistant BC cells, raises the possibility that targeting both proteins at once with specific inhibitors could represent a new strategy against BCs and overcoming high dose side effects and modest efficacy in patients exposed to single doses of Dot1L inhibitors [47]. The effects of EPZ alone on proliferation and death in a panel of luminal-like BC cells, including MCF-7, have been already evaluated in detail [8], and the results obtained here are in agreement with our previous findings. This compound induced a concentration-dependent inhibition of cell growth in AE-sensitive (MCF7), tamoxifen (TAM-R) or fulvestrant/ICI (ICI-R)-resistant MCF-7 cells (Fig. 2D–F; upper panels). The same strong inhibition of AE-sensitive and AE-resistant BC cell growth was observed also upon menin inhibition by MI-136 [20] (Fig. 2D–F; lower panels). Of note, these results confirmed the gene response elicited by its analog (MI-2) on MCF-7 cell transcriptome (Fig. 2A). A combination of EPZ and MI-136 was then tested in all three cell lines and additionally on other luminal-like BC cell models (BT474, T47D and ZR75.1; Additional file 1: Fig. S3C–E), to evaluate a possible combinatory effect of the two inhibitors. Interestingly, the test revealed a synergistic effect of the two drugs on cell growth, with a very significant growth inhibition observed in all luminal-like BC cell lines tested at suboptimal concentrations of both drugs (Fig. 2G–I and Additional file 1: Fig. S3C–E). This result was confirmed with MI-503, another menin pharmacological inhibitor derived from the MI-136 scaffold [21] (data not shown), or with siRNA-mediated (Additional file 1: Fig. S3F–H) DOT1L and MEN1 silencing (Additional file 1: Fig. S3I–K). The growth inhibitory effects of EPZ and MEN1, either alone or in combination, are accompanied by down-regulation of ERα expression (Fig. 2J–L), an effect that is likely to contribute to the antiproliferative effects of these compounds in estrogen-responsive BC cells [8, 48]. Even in this case the combined treatment with EPZ and MI136 resulted in a more pronounced down-regulation of ESR1 mRNA that either compound used alone. These results, further suggest that Dot1L and menin act on common targets in AE-sensitive and AE-resistant ER + luminal-like BC cells.

### Mapping Dot1L and menin nuclear interactomes identifies BAZ1B as a new partner of these proteins in chromatin remodeling complexes

**Fig. 3:**
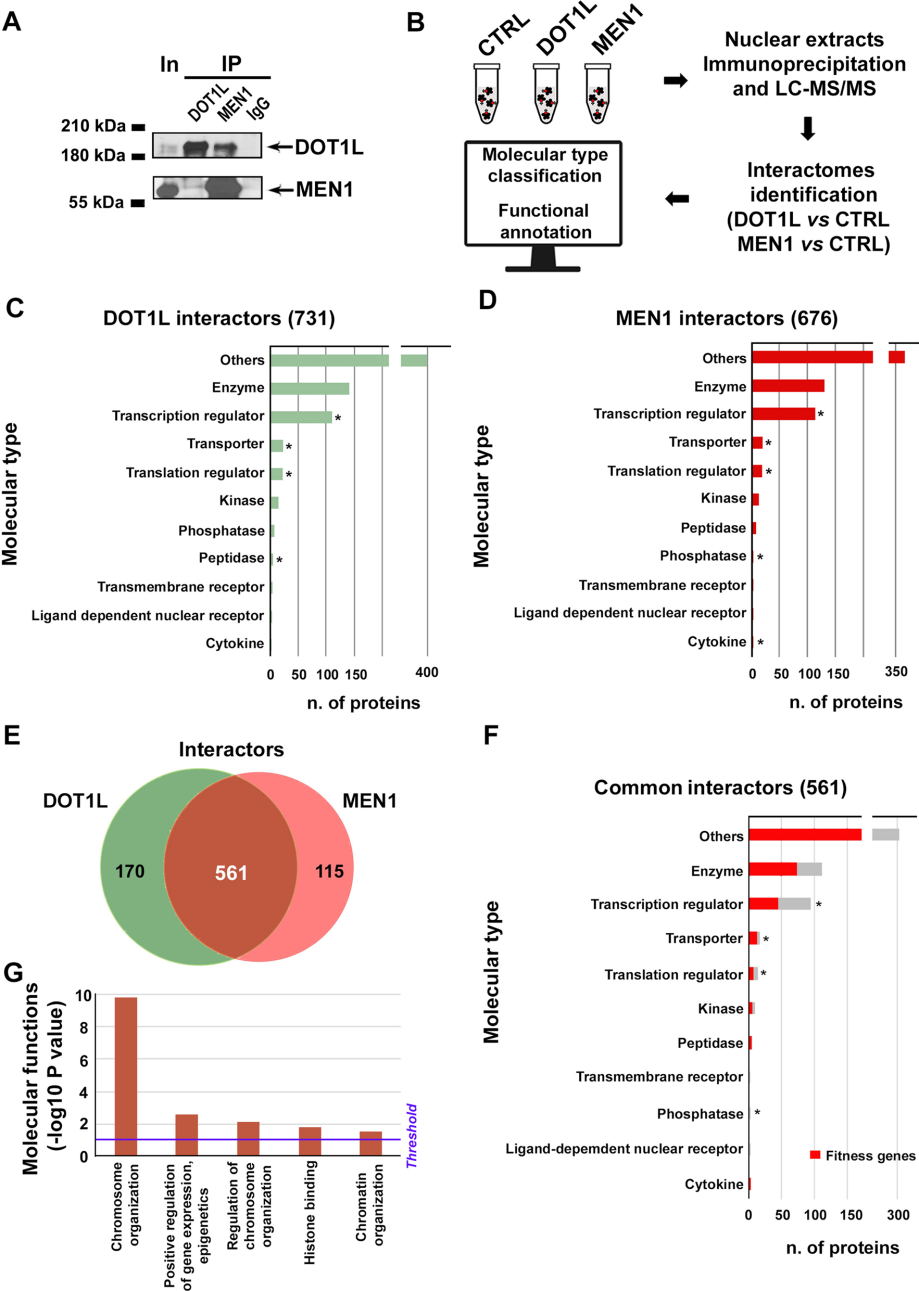
Dot1L and menin interactomes analysis in MCF-7 BC cell nuclei. **A** Representative western blot showing Dot1L and menin co-immunoprecipitation in MCF-7 nuclear extracts. IgG was used as negative control. **B** Experimental workflow for Dot1L and menin interactome identification and characterization. For each sample three independent biological replicates were performed. (**C** and **D**) Bar charts showing molecular type classification of Dot1L (left, green) and MEN1 (right, red) associated proteins. Asterisks indicate statistical significance (p* ≤ 0.05). **E** Venn diagram showing overlaps between Dot1L and MEN1 associated interactors. **F** Bar chart showing the molecular type classification of the 561 Dot1L andMEN1 common interactors. The red line indicates the number of interactors that are also fitness genes. Asterisks indicate statistical significance (p* ≤ 0.05). **G** Results of IPA (Ingenuity Pathway Analysis) functional analysis showing statistically significant molecular functions enriched in Dot1L and menin common interactors

**Fig. 4:**
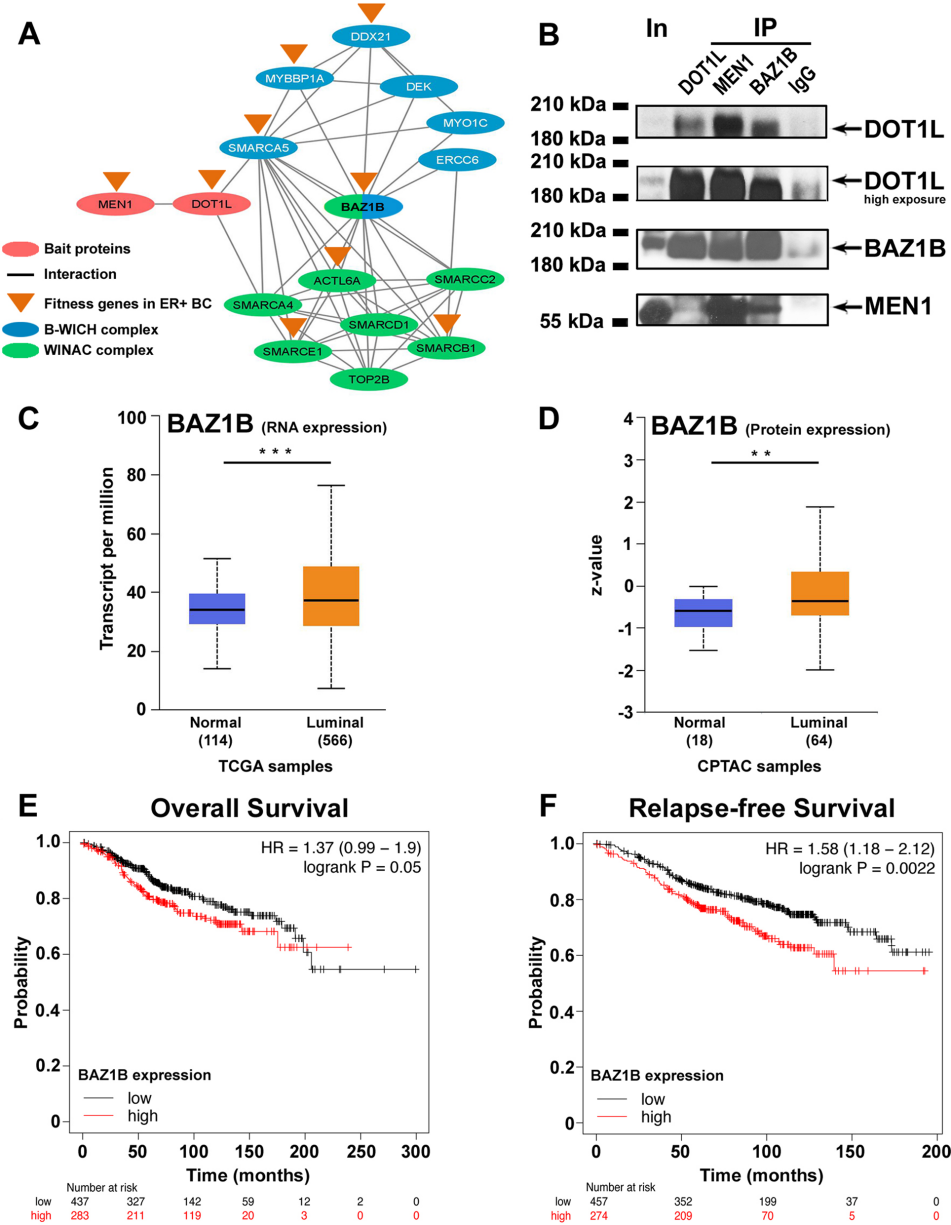
Association between BAZ1B, Dot1L and menin and analysis of BAZ1B expression in BC tumors. **A** Network reprentation interaction between Dot1L, menin, BAZ1B and the proteins of B-WICH (blue) and WINAC (green) complexes found among Dot1L and menin common interactors. **B** Western blot showing BAZ1B, Dot1L and menin co-immunoprecipitation MCF-7 nuclear extracts. BAZ1B mRNA **C** and protein **D** expression levels in luminal-like breast cancer samples from The Cancer Genome Atlas (TCGA) and Clinical Proteomic Tumor Analysis Consortium (CPTAC) datasets analyzed with UALCAN. Kaplan–Meier curves, generated using the Kaplan–Meier Plotter, showing the probability of overall (**E**; 437 low and 283 high samples, respectively**)** and relapse-free (**F**: 457 low and 274 high samples, respectively**)** survival of ERα + BC patients according to BAZ1B mRNA expression levels

Regulatory protein activity is known to involve several partners, assembled in multiple complexes, often unique to specific cellular compartments, comprising different molecular partners each endowed with distinct functions, according to the principles of combinatorial chemistry. Mapping a regulatory factor interactome can, therefore, provide useful clues to understand its multifaceted effects in a specific cell type. ERα interactomes in luminal-like BC cells are indeed different depending upon the cellular compartment (nucleus or cytoplasm) or, in the nucleus, the nature of the ligand [10, 34, 49]. The same applies for the nuclear interactome of estrogen receptor beta (ERβ), that is quite different in luminal-like compared to triple-negative BC cells [26, 27, 50]. To explore the molecular mechanism underlying Dot1L and menin activities on the BC cell genome, and the basis of the synergistic effects of their inhibitors on cell growth, the nuclear interactomes of the two proteins were identified, characterized and annotated in AE-sensitive MCF-7 cells by interaction proteomics, as summarized in Fig. 3A, B. To this aim, native nuclear complexes were first immunoprecipitated with anti-Dot1L, anti-menin or, as control, nonspecific IgGs antibodies, and then subjected to MS analysis. Three independent biological replicates for each experimental condition were analyzed, in parallel, and principal component analysis was performed as control, showing a good correlation between the biological replicates of each condition (Additional file 1: Fig. S4A). Efficiency of the IP procedure was also confirmed by comparing signal intensity values relative to Dot1L and menin in each sample vs controls (Additional file 1: Fig. S4B). This analysis identified 731 and 676 nuclear proteins co-purified with Dot1L and menin, respectively, among which prevailed enzymes, transcription and translation regulators and transporters (Fig. 3C, D and Additional file 3: Table S2). Functional enrichment analysis performed by IPA, revealed involvement of Dot1L and menin interactors in multiple cellular processes, such as gene expression, cell cycle, cell death and survival, cell growth and proliferation, highlighting a specific involvement of Dot1L and its molecular partners in the control of cellular movement, protein degradation and protein folding (Additional file 1: Fig. S4C). Interestingly, comparative analysis of the two interactomes showed a significant similarity of the two protein sets. As shown in Fig. 3E, the majority (561) of the interactors were in common between the two factors (Additional file 4: Table S3), many encoding enzymes or transcriptional regulators (Fig. 3F). Of note, 60% of them are BC fitness genes, essential for proliferation and survival of cancer cells [51]. Functional annotation analysis focusing on the 561 common components of the Dot1L and menin interactomes indicates that these are involved in key molecular processes related to gene transcription and genome structure and activity, such as epigenetic regulation of gene expression, histone binding and chromosome and chromatin organization (Fig. 3G), all taking part in the reprogramming of chromatin landscape that has been recognized as a critical step of the transcriptional response in breast [52] and other cancers. Due to the relevance of chromatin organization and its deregulation in BC, we mined the proteome data for protein subnetworks known to be directly involved in this process. This analysis, performed on the 561 interactors in common between Dot1L and menin (Additional file 5: Table S4), revealed the presence of several components of the B-WICH and WINAC chromatin remodeling complexes. Interestingly, a bromodomain protein encoded by the Bromodomain Adjacent To Zinc Finger Domain 1B gene (BAZ1B), also known as WSTF (Williams Syndrome Transcription Factor), resulted as one of the central components of both complexes (Fig. 4A) [53]. Given the role of bromodomain proteins as ‘readers’ of epigenetic marks that translates these signals into gene regulatory events, the interaction between Dot1L, menin and BAZ1B was experimentally validated by co-IP experiments in nuclear lysates from MCF-7 cells (Fig. 4B). Analyzing TCGA and CPTAC BC datasets, we observed that BAZ1B mRNA and protein are expressed at higher levels in luminal-like BC respect to normal mammary gland (Fig. 4C, D) and, more interestingly, higher BAZ1B expression was associated with worse overall and relapse-free survival in ER + BCs (Fig. 4E and F, respectively). These results suggest the possibility that, within the Dot1L-menin network identified here, BAZ1B might represent an additional actionable target against endocrine therapy-resistant BC.

### BAZ1B silencing disrupts estrogen signaling in AE-sensitive and AE-resistant BC cells

**Fig. 5:**
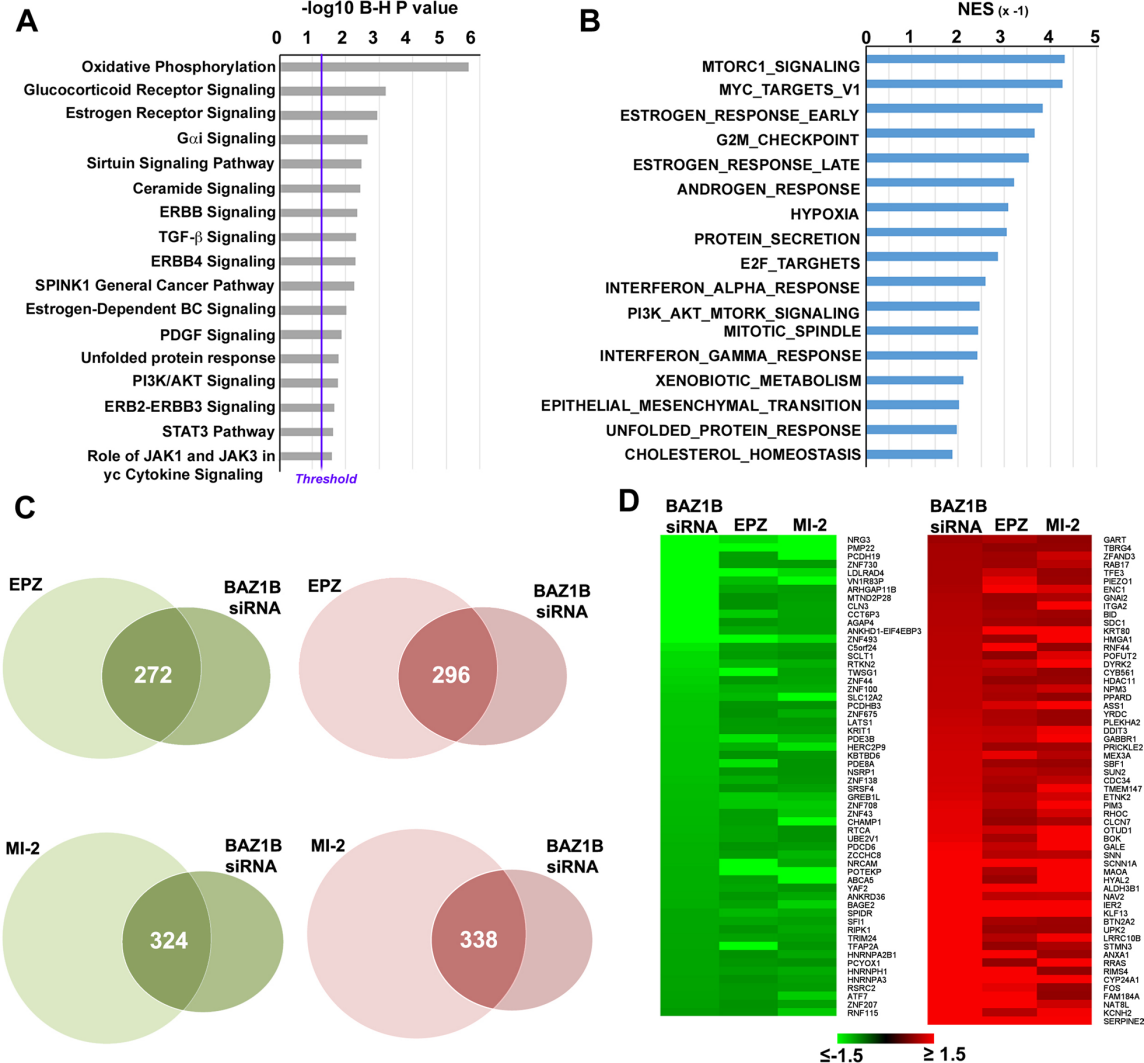
Impact of BAZ1B silencing on gene expression in MCF-7 BC cells. **A** Results of IPA (Ingenuity Pathway Analysis) functional analysis showing statistically significant pathways enriched following BAZ1B silencing in MCF-7 cells for 72 h. **B** Statistically significant functions highlighted by Gene Set Enrichment Analysis (GSEA) in siRNA-mediated BAZ1B kd cell transcriptome in MCF-7 cells. NES: Negative Normalized Enrichment Score. **C** Venn diagram showing the number of common transcripts up-regulated (red) and down-regulated (green) following EPZ treatment or siRNA-mediated BAZ1B kd (upper panel) and MI-2 treatment or siRNA-mediated BAZ1B kd (lower panel) in MCF-7 cells. **D** Heatmap showing common down-regulated (green) and up-regulated (red) transcripts following BAZ1B silencing, EPZ or MI-2 treatments in MCF-7 cells

**Fig. 6:**
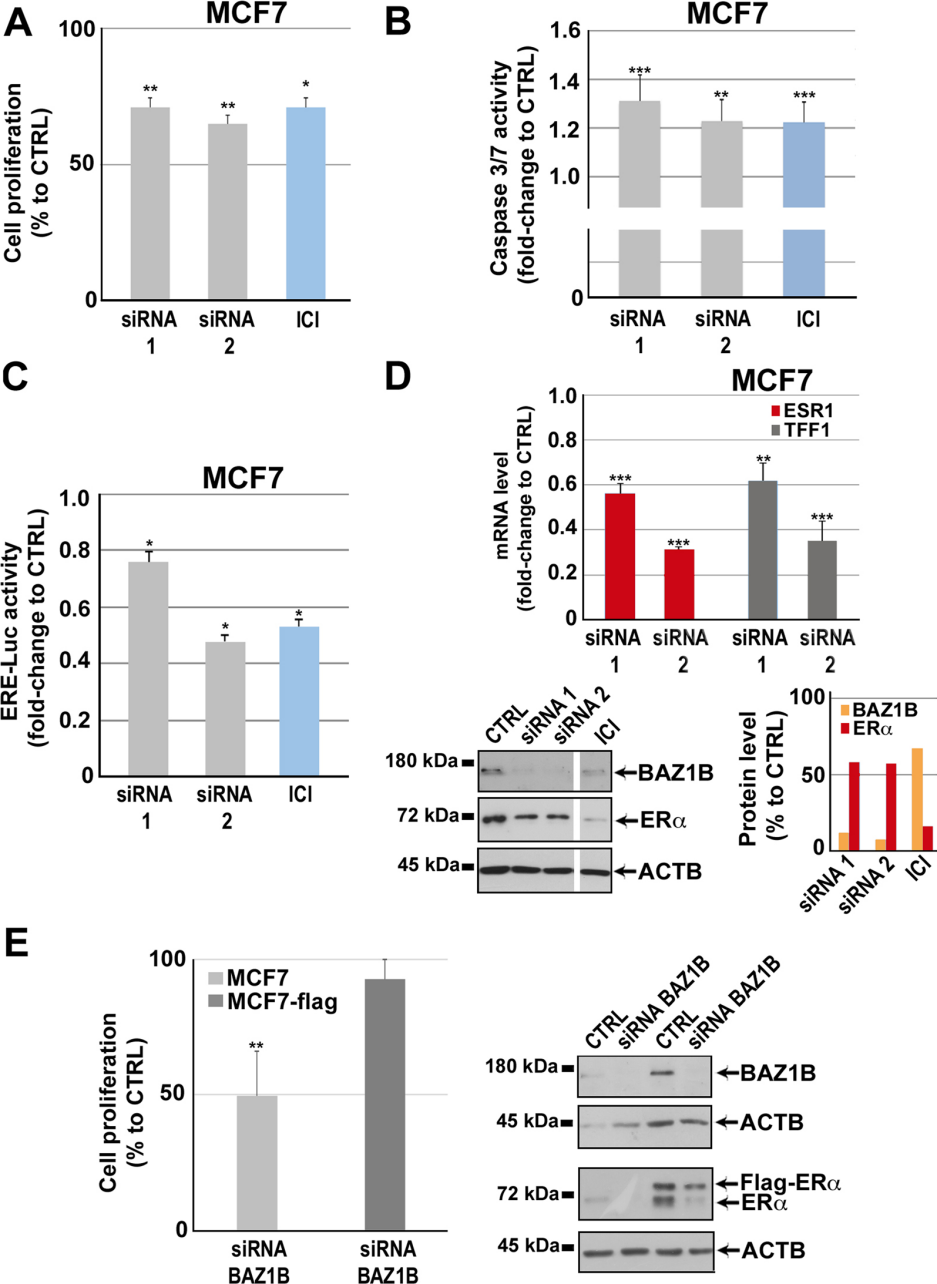
Effect of BAZ1B silencing in MCF-7 BC cells. MCF-7 cell proliferation rate **A** and Caspase 3/7 activity assay **B** performed following BAZ1B silencing or treatment with fulvestrant (ICI; 100 nM). **C** ERα trans-activating activity assessed in MCF-7 cells stably expressing the ERE-Luc reporter gene following BAZ1B silencing or treatment with ICI (100 nM). All data are analyzed respect to the scramble (Silencer Select Negative Control: CTRL). Data are presented as the mean ± SD of determinations from a representative experiment performed in six independent replicates after 72 h of silencing. **D** RT-qPCR (upper panel) analysis of ESR1 (ERα) and TFF1 mRNA levels following BAZ1B silencing. RT-qPCR results are shown as mean ± SD of triplicate determinations from a representative experiment after 72 h of silencing. Western blot (left lower panel) and relative densitometry (right lower panel) showing BAZ1B and ERα protein level following BAZ1B silencing or treatment with ICI (100 nM). β-actin (ACTB) was used as control. Images were processed with ImageJ software (https://imagej.net) for densitometry readings. **E)** Cell proliferation rate (left) and western blot analysis (right) comparing MCF-7 and MCF-7-flag cells following BAZ1B silencing. For MTT assay, all data are analyzed respect to the scramble (Silencer Select Negative Control: CTRL). Data are presented as the mean ± SD of determinations from a representative experiment performed in six independent replicates after 72 h of silencing. Western blot analysis showing BAZ1B and ERα protein level following BAZ1B silencing. β-actin (ACTB) was used as control. Asterisks indicate statistically significant differences (*p ≤ 0.05, **p ≤ 0.01, ***p ≤ 0.005) to CTRL

To investigate the functional role of BAZ1B in BC cell nuclei, exponentially growing MCF-7 BC cell cells were subjected to BAZ1B kd by transient transfection of two siRNAs, targeting different regions of the mRNA and both able to reduce by 70–80% the level of this mRNA (Additional file 1: Fig. S5A). As shown in Additional file 1: Fig. S5B, silencing BAZ1B for 72 h, with either siRNA employed, determined a significant effect on MCF-7 cells transcriptome, including 841 transcripts affected (541 down- and 300 up-regulated, compared to control siRNA-treated cells; fold change ≤ and ≥|1.5|). Transcriptome changes induced by BAZ1B silencing highlighted an effect on oxidative phosphorylation, mediated by changes in several nuclear-encoded mitochondrial genes (data not shown), and on signal transduction pathways known to control several key features of BC cells, including those controlled by estrogen (Fig. 5A). Transcriptome data analysis by GSEA confirmed and expanded these results, showing a significant effect not only on early and late estrogen response gene pathways, but also on those related to myc, mTORC, PI3K, AKT signaling, to cell cycle checkpoints and to epithelial-to-mesenchymal transition (Fig. 5B). Interestingly, when comparing the effects of BAZ1B kd on the cell transcriptome to those elicited by pharmacological inhibition of Dot1L or menin with EPZ and MI-2, respectively, 569 transcripts (272 down-regulated and 296 up-regulated) showed a similar response in the case of EPZ and 662 (324 down-regulated and 338 up-regulated) for MI-2 (Fig. 5C). Considering the top regulated transcripts in common between the three conditions (123, 61 up-regulated and 62 down-regulated; fold change ≤ and ≥|1.2|), these encoded several transcriptional and epigenetic regulators and signal transducers known to regulate cell growth (Fig. 5D), Indeed, BAZ1B silencing reduced significantly MCF-7 cell proliferation already within the first 72 h from siRNA transfection (Fig. 6A), accompanied by activation of apoptosis (Fig. 6B), to an extent comparable to that induced by ICI. A significant inhibition of cell growth was also observable in additional ERα + BC cell models (BT474, T47D and ZR75.1) as shown in Additional file 1: Fig S6A–F. Of note, since estrogen is the main growth stimulus for these luminal-like BC cells, these data, combined to the finding that estrogen signaling results inhibited under these conditions, also from transcriptome analysis following BAZ1B kd (Fig. 5A, B), suggested us that activity of this protein could be required, here, to allow ERα signaling. Indeed, as shown in Fig. 6C, D BAZ1B silencing prevents efficient receptor-mediated transactivation of the reporter gene ERE-luc and of its well-known target gene TFF1 [54], mainly by strongly down-regulating ERα mRNA and protein. A marked decrease of estrogen signaling upon reduction of the cellular levels of BAZ1B was confirmed here by comparing the gene expression changes measured under these conditions to those induced by ERα inhibition with ICI, revealing 395 transcripts equally influenced by the two conditions (229 up-regulated and 166 down-regulated, data not shown). To confirm our hypothesis we restored ESR1 expression using exogenous full-length-3xFlag-ESR1 (ERα) in MCF-7 (MCF-7-flag) cells and compared the ability of BAZ1B silencing to inhibit cell proliferation and disrupt ERα protein level in this cellular model with respect to MCF-7 cells. Results of this ‘complementation’ test reported in Fig. 6D confirmed the hypothesis of a direct involvement of the receptor in mediating BAZ1B effects. Indeed, both expression of the exogenous ERα and proliferation of cells expressing it were not affected by BAZ1B *kd*.

**Fig. 7:**
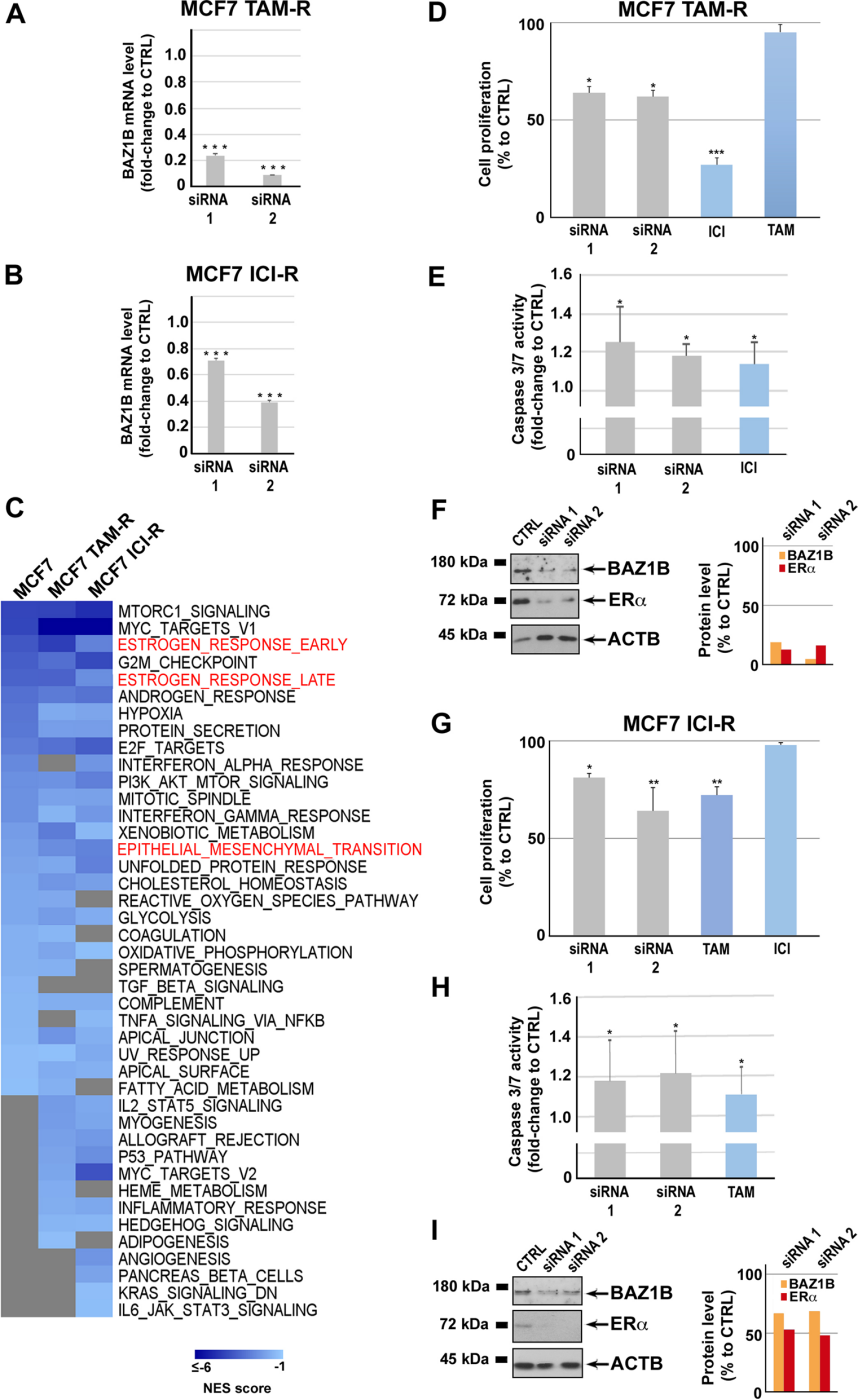
Effects of BAZ1B silencing in antiestrogen-resistant BC cells. RT-qPCR analysis of BAZ1B mRNA level following BAZ1B silencing in tamoxifen (MCF7 TAM-R; **A**)- and fulvestrant/ICI (MCF7 ICI-R; **B**)-resistant BC cells. RT-qPCR results represent mean ± SD of triplicate determinations from a representative experiment after 72 h of silencing. Asterisks indicate statistically significant differences (***p ≤ 0.005) to CTRL. **C** Heatmap showing the Normalized Enrichment Score (NES) of selected functions involving gene expression changes in AE-sensitive (MCF7), tamoxifen (MCF7 TAM-R**)-** or fulvestrant/ICI (MCF7 ICI-R**)**-resistant BC cells following BAZ1B silencing. Key functions are highlighted in red. Cell proliferation rate assessed by MTT assay in tamoxifen (MCF7 TAM-R; **D**)- and fulvestrant/ICI (MCF7 ICI-R; **G**)-resistant BC cell lines following BAZ1B silencing and treatment with ICI (100 nM) or tamoxifen (TAM; 100 nM). Caspase 3/7 activity assay performed in the same experimental condition mentioned above in tamoxifen (MCF7 TAM-R; **E**)- and fulvestrant/ICI (MCF7 ICI-R; **H**)-resistant BC cells. All data are analyzed respect to the scramble (Silencer Select Negative Control: CTRL). Data are presented as the mean ± SD of multiple determinations from a representative experiment performed six independent replicates after 72 h of silencing. Asterisks indicate statistically significant differences (*p ≤ 0.05, **p ≤ 0.01, ***p ≤ 0.005) to CTRL. Western blot and relative densitometry showing ERα protein level in tamoxifen (MCF7 TAM-R; **F**)- and fulvestrant/ICI (MCF7 ICI-R; **I**)-resistant BC cells following BAZ1B silencing. β-actin (ACTB) was used as loading control. Images were processed with ImageJ software (https://imagej.net) for densitometry readings

The impact of BAZ1B on ERα activity in hormone-responsive BC cells suggested the hypothesis that this protein could be an exploitable target to disrupt ERα-mediated signaling also in AE-resistant BC models that recapitulate the endocrine therapy-resistant tumor phenotype [55]. We therefore tested the effects of BAZ1B silencing (Fig. 7A–B and Additional file 1: Fig. S7) on growth and transcriptome of TAM-R and ICI-R cells. As shown in Fig. 7C, GSEA analysis of the gene expression changes detected under this conditions revealed an extensive transcriptome reprogramming in both cell lines, with the majority of the changes affecting the same cell signaling and functional pathways found in AE-sensitive MCF-7 cells under the same experimental conditions (Fig. 5B). In line with these results, BAZ1B kd in TAM-R and ICI-R cells resulted in reduction of cell proliferation (Fig. 7D and G), increased apoptosis (Fig. 7E and H) and dowregulation of ERα mRNA and protein (Fig. 7F and I) to an extent comparable to what observed previously in AE-sensitive cells (Fig. 6).

**Fig. 8:**
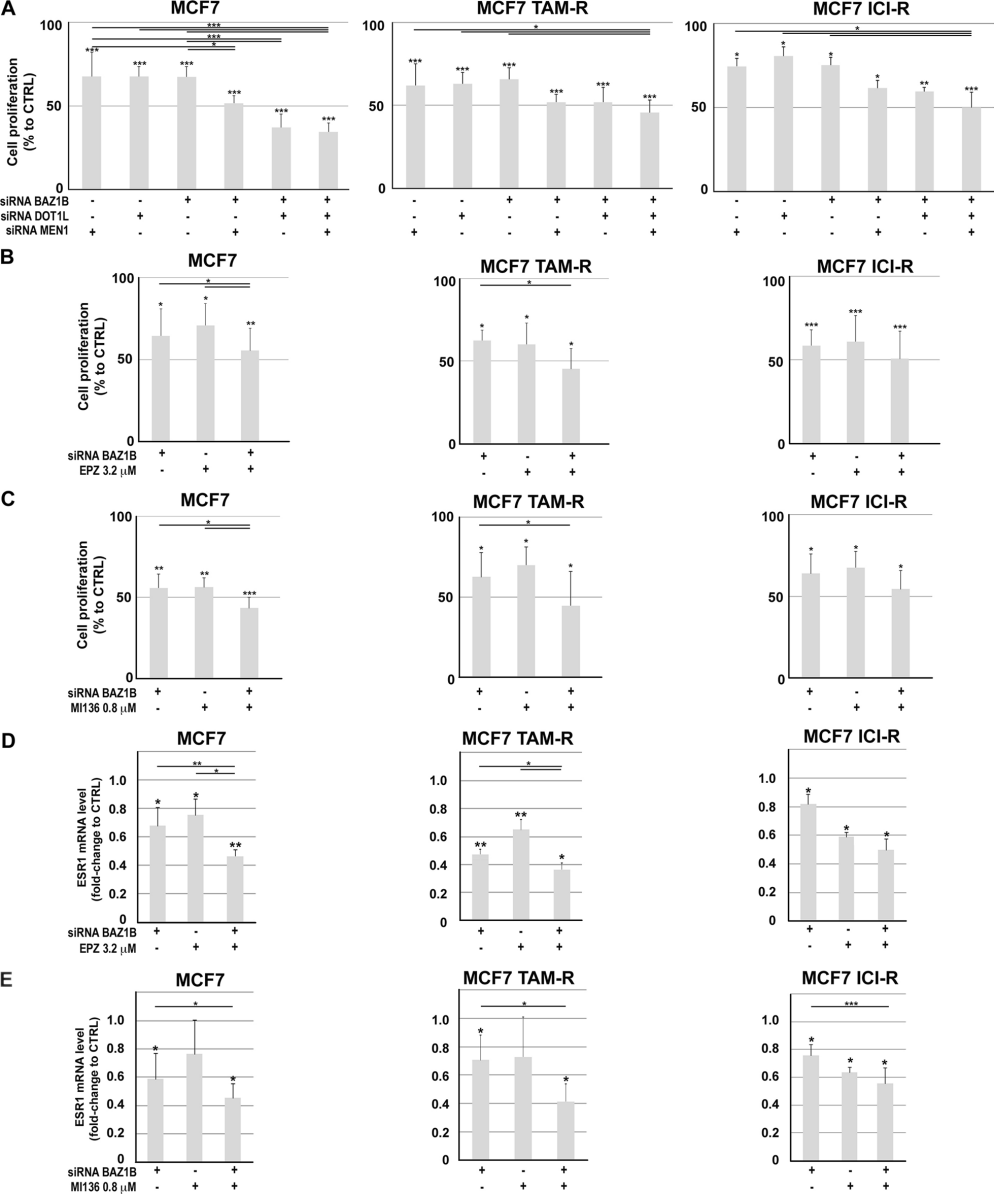
Effects of BAZ1B silencing in combination with Dot1L or menin blockade in antiestrogen-sensitive and antiestrogen-resistant BC cells. **A** Proliferation rate assessed by MTT assay in AE-sensitive (MCF7) tamoxifen (MCF7 TAM-R**)**- and fulvestrant/ICI (MCF7 ICI-R**)**-resistant BC cells following BAZ1B, Dot1L or menin silencing or combining BAZ1B silencing with EPZ **B** or MI-136 **C** treatment. Data are presented as the mean ± SD of multiple determinations from a representative experiment performed in multiple replicates. RT-qPCR showing ESR1 (ERα) mRNA level by combining BAZ1B silencing with EPZ **D** and MI-136 **E** pharmacological inhibition in the same experimental condition indicated above. RT-qPCR results represent mean ± SD of triplicate determinations from a representative experiment. Asterisks indicate statistically significant differences (*p ≤ 0.05, **p ≤ 0.01, ***p ≤ 0.005) to CTRL or to single treatment (black bars)

The transcriptome changes resulting from their inhibition in the luminal-like BC cell models used here strongly suggest that BAZ1B, Dot1L and menin exert both specific and common effects on gene transcription leading among other to a genetic control of mitogenic pathways, comprising, in particular, those dependent on estrogen signaling via ERα, supported by the fact that ERα + BC characterized by concomitant high levels of BAZ1B, DOT1L and MEN1 genes exhibited a worst overall and relapse-free survival (Additional file 1: Fig S8), resembling more aggressive tumors generally resistant to endocrine therapy regimens. Since the interaction proteomics summarized in Fig. 4A suggest that all three these nuclear factors may act in concert within the cell nucleus as subcomponents of the B-WICH and WINAC chromatin remodeling complexes, and those deriving from kd and/or pharmacological inhibition of each of them were in agreement in showing a marked effect of cell growth, survival and ERα levels in the cell, we investigated whether the contemporary inhibition of either two or all three of them in AE-sensitive and AE-resistant cells would result in more pronounced effect on proliferation and receptor expression. As shown in Fig. 8A, combined siRNA silencing of BAZ1B with either Dot1L or menin or all together enhanced their inhibitory effects on cell growth in all three cell lines, with a stronger reduction in cell number, respect to control siRNA, detectable already within the first 72 h of treatment. A comparable result was obtained when BAZ1B kd was combined with a suboptimal concentration of EPZ (Fig. 8B) or MI-136 (Fig. 8C). Interestingly, once again the growth inhibitory effects were mirrored in all conditions by down-regulation of ERα mRNA (Fig. 8D–E). Taken together, these results indicate that the antiproliferative effects of BAZ1B, Dot1L and menin in ER + BC cells are in all cases directly linked to inhibition of estrogen signaling by clearing ERα form the cell. Since also TAM- or ICI-resistant cells respond in the same way, these results are concordant in suggesting the possibility to target these three factors, either alone or in combinations, with specific inhibitor in new therapeutic regimens against endocrine therapy-resistant breast tumors that still express wt or mutated ERα, to achieve an antiestrogen-like effect due to lower receptor levels that, in turn, cause reduction of cell proliferation and increased cell death.

## Discussion

Endocrine therapy represents the first line pharmacological regimen for ERα + BCs, although in most cases it results are very positive for the patient, it is ineffective in about 30% of cases, due to intrinsic and acquired resistance of cancer cells to the antiestrogenic activity of the drugs used, resulting in severe consequences due to tumor relapse and metastasis. Resistance to hormonal therapy of the tumors expressing ERα is thought to be caused by different genetic and epigenetic mechanisms, including mutations of the receptor itself or of key components of its signal transduction pathway to the cell genome that make it constitutively active or insensitive to AE ligands [4]. Among the functional partners of ERα, those that act in the nucleus in concert with it to convey the estrogenic signal to the transcriptional machinery belong to multiprotein complexes including, among others, epigenetic and chromatin remodeling factors. Considering that all these proteins collaborate with each other toward an effective functioning of the ERα-associated gene regulatory machinery, it is possible to imagine that multiple components of these complexes can be targeted, either alone or in combination, to inhibit their activity resulting in all cases in disruption of ERα activity on gene expression and, as a consequence, tumor growth and progression Combinational targeted therapy represents a useful and much sought after approach for cancer therapy and, in this context, epigenetic pharmacology based on ‘epidrugs’ influencing the activity of specific vulnerabilities in cancer cells represent a promising approach against resistance to current therapeutic regimens [5, 56].

Toward this aim, we analyzed here in detail the functional interplay between Dot1L and menin in luminal-like ER + BC cells both sensitive and resistant to antiestrogens. We demonstrated that Dot1L is associated with menin in BC cell chromatin and that both proteins co-localize in a functionally relevant fraction of genomic sites, half of them shared with ERα. This physical and functional interrelationship between the two epigenetic regulators was confirmed by analysis of the effects of their pharmacological inhibition on the cell transcriptome. This demonstrated that these two factors target in many cases the same cellular pathways and processes, including in particular early and late response to estrogens. Although targeting Dot1L or menin alone is a promising strategy for treating breast and other cancers [8, 10, 20, 21, 48, 57, 58], the results of early phase clinical trials revealed modest efficacy and possible side effects in patients exposed to a single agent, suggesting the need for optimized strategies, including also combination therapies [47, 59]. On this line, we sought out to characterize the effects of combined inhibition of Dot1L and menin in BC cell models of AE-sensitive and AE-resistant BC. The result obtained clearly demonstrate that combined pharmacological inhibition of these two targets is indeed very effective in reducing ERα expression and estrogen signal transduction, and therefore a promising strategy to try to overcome resistance to endocrine therapy in ERα + tumors. Interestingly, our results suggest that this strategy is very effective even when using lower doses of each inhibitor, while still retaining specificity. It is worth mentioning that a Dot1L and menin combinatory inhibitors approach has been already proposed for therapy of some hematological malignancies including NPM1-mutant [60] and MLL-rearranged [61] leukemias. Results proposed in these studies support our findings expanding the potential of combinatory therapy targeting these molecules in solid tumors, where single agents have been already proven to be not fully effective and safe.

Investigating in detail the functional correlations between DOT1L and menin by interaction proteomics we identified a sizable number of molecular partners shared by them. Interestingly, these include many essential genes for BC cell proliferation and survival [51, 62], including several involved in key processes in hormone-responsive BC cells. These include, among others, known ERα interactors [8, 63] and chromatin remodeling proteins, including several involved in B-WICH, a transcription regulator complex, and WINAC, a chromatin remodeling complex for replication. Among them, BAZ1B caught our attention being shared by both these complexes and associated with both Dot1L and menin on chromatin. BAZ1B is a versatile nuclear protein that, in addition to functioning as chromatin remodeler, has a protein tyrosine kinase activity [53] and has been involved in DNA replication and repair and transcriptional regulation. An oncogenic role of BAZ1B in cancers has been postulated via its phosphorylation and acetylation, where it has been proposed that its abnormal activity would favor cancer progression [53]. Given the crucial role of chromatin accessibility and remodelers in endocrine resistance in BC [55], we characterized in detail the role of BAZ1B and the effects of its inhibition in the breast tumor subtype that express ERα. Involvement of this protein in estrogen-dependent signaling clearly emerged by transcriptome profiling, whereby BAZ1B kd was found to mimick the effects of antiestrogens on cell proliferation and death, through a direct impact on receptor expression and, consequently, its target genes. Of interst, luminal-like breast tumors characterized by high levels of BAZ1B exhibited a worst overall and relapse-free survival, reflecting the more aggressive phenotype of these tumors, generally refractory to AE therapies. Thus, targeting this protein to overcome failure of endocrine therapies has, in our opinion, clinical relevance and for this reason it was tested in AE-resistant BC models by siRNA-mediated silencing, since inhibitors of this proteins are not available yet. The results obtained recapitulated the effects observed in AE-sensitive cells and were found to be mediated, also here by estrogen desensitization and ERα down-regulation. Interestingly, combining siRNA-mediated silencing of BAZ1B with silencing or pharmacological inhibition of Dot1L and menin resulted in more pronounced reduction of cell proliferation, coupled with an increased effect in ERα clearing, in both AE-sensitive and AE-resistant BC cells. Since aberrant activity of the receptor or its partners is considered the main cause of endocrine resistance [55], this result is of particular interest as it shows a new potential approach to effectively interfere with ERα signaling in cancer cells by targeting the complex formed by the three factors investigated here.

The estrogen receptor way of action resembles, in this context, those elicited by dependence receptor controlling major aspects of tumorigenesis, such as proliferation, angiogenesis, invasiveness and resistance to therapeutic regimens [64]. Much like inhibiting dependence receptors represents a very promising approach toward more effective targeted cancer therapy, shutting down ERα in the cell is undoubtedly a rationale and effective way against endocrine-resistant and metastatic tumors. Dot1L, menin and BAZ1B are functional to this aim, also when considering the possibility of a combinatorial targeting of two or all three these factors. In this respect, it is worth mentioning that BAZ1B bromodomain motive and atypical protein tyrosine kinase activity [53] are both prone to be targeted by small molecule inhibitors, for a novel pharmacological strategy to block this protein and thereby act on ESR1 expression.

We envision the combinatorial therapy approach proposed here could represent a novel strategy to improve treatment of ER + BCs, in particular those resistant to endocrine therapy, including it in the therapeutic protocols presently adopted (AE, AI and CDK4/6 inhibitors), could help maximize the therapeutic efficacy. Indeed, the combined use of CDK4/6 inhibitors (e.g., palbociclib, ribociclib, abemaciclib) and AEs have markedly prolonged progression-free survival, compared with AE alone, in patients with ER + metastatic BC. Therefore, targeting the components of the functional complex described here with a combination of the already available drugs could help improve the therapeutic response in tumor recurrences as well as early-stage cancers.

## Conclusions

In conclusion, the results reported here provide new insights on the functional interplay between ERα, Dot1L, menin and BAZ1B in endocrine therapy-sensitive and therapy-resistant BC. Although requiring additional research concerning the role and mechanism of action of BAZ1B in this context, these data reveal a new therapeutic vulnerability of endocrine therapy-resistant cancer cells, demonstrated by the effects of inhibition of the three factors on ERα expression and multiple key pathways essential for their growth, survival and invasion.

## Supplementary Information

**Additional file 1: Fig. S1.** Dot1L and menin expression in breast cancer. Dot1L (**A**, left panel) and menin (**A**, right panel) mRNA levels in normal and luminal breast cancer samples from The Cancer Genome Atlas (TCGA), analyzed with UALCAN. **B**) Dot1L and menin mRNA co-expression from two additional BC patient datasets from TCGA. Only expression values above the first quartile were considered. **Fig. S2.** Dot1L, menin and ERα binding to MCF-7 cell chromatin. Heatmap (left panel) showing read density within 10 kb regions centred on Dot1L, menin and ERα binding sites in MCF-7 cells. Control (CTRL) was obtained using nonspecific Abs. Overrepresented transcription factor binding motifs within Dot1L+menin+ERα binding sites (green) or Dot1L+menin binding sites (blue) are reported in the word cloud (right panel). **Fig. S3.** Impact of Dot1L and menin blockade on gene expression and cell proliferation in BC cells. Bar charts from KEGG functional enrichment analysis showingstatistically significant pathways deregulated upon MCF-7 cell treatment with EPZ (**A**) or MI-2 (**B**). Combenefit software was used to generate dose-response surface curves in BT474 (**C**), T47D (**D**) and Zr75.1 (**E**) BC cells according to **D**–**R** Lowe model, combining increasing dose of EPZ and MI-136 for 9 days. Color scale bar indicate the level of synergy (blue) or antagonism (red) at each combination. Effect of siRNA-mediated Dot1L and menin silencing on AE-sensitive (MCF7; **F**) and tamoxifen (MCF7 TAM-R; **G**)- or fulvestrant/ICI (MCF7 ICIR; **H**)-resistant BC cells assessed by RT-qPCR (top) and western blot (middle) assays. RT-qPCR results are shown as mean ± SD of triplicate determinations from a representative experiment after 72h of silencing. Western blot show Dot1L or menin protein levels following their silencing. β-actin (ACTB) was used as control. Effect of siRNA-mediated Dot1L and menin silencing on AE-sensitive (MCF7; **I**) and tamoxifen (MCF7 TAM-R; J)- or fulvestrant/ICI (MCF7 ICIR; **J**)-resistant BC cells assessed on cell proliferation. MTT data are presented as the mean ±SD from six independent replicates after 72 h of silencing. Asterisks indicate statistically significant differences (*p ≤0.05, **p ≤0.01, ***p ≤0.005) to CTRL or to single treatment (black bars). **Fig. S4.** Technical and functional evaluation of Dot1L and menin interactome analysis by mass spectrometry. **A** Principal component analysis (PCA) of the mass spectrometry (MS) results obtained from three biological replicates of control (CTRL; IgG: blue), Dot1L (red) and menin (MEN1; green) samples. Numbers between parentheses indicate percentage of total variance. **B** Box plot showing Dot1L (red) and menin (green) protein levels detected in CTRLs and samples by MS. Asterisks indicate statistically significant differences (*p ≤0.01). **C** Results of IPA (Ingenuity Pathway Analysis) functional analysis showing statistically significant molecular functions enriched in Dot1L (green) and menin (red) interactomes. **Fig. S5.** Impact of BAZ1B silencing on gene expression in MCF-7 BC cells. **A** RT-qPCR analysis of BAZ1B mRNA level after silencing with two different siRNAs compared to scramble (Silencer Select Negative Control: CTRL). RT-qPCR results shown are the mean ± SD of multiple determinations from a representative experiment. Asterisks indicate statistically significant differences (***p ≤0.005). **B** Heatmap showing top down-regulated (green) and up-regulated (red) transcripts, fold change ≤ and ≥ |1.5|, following BAZ1B silencing with both siRNAs, compared to scramble (Silencer Select Negative Control: CTRL). **Fig. S6.** Effect of BAZ1B silencing in luminal-like BC cell models. RT-qPCR (upper panel) and western blot analysis of BAZ1B mRNA and protein levels following BAZ1B silencing in BT474 (**A**), T47D (**B**) and ZR75.1 (**C**) BC cells. RT-qPCR results are shown as mean ± SD of triplicate determinations from a representative experiment after 72 h of silencing. Western blot shows BAZ1B protein level following BAZ1B silencing. β-actin (ACTB) was used as control Cell proliferation rate performed following BAZ1B silencing or treatment with fulvestrant (ICI; 100 nM) in BT474 (**D**), T47D (**E**) and ZR75.1 (**F**) BC cells. All data are analyzed respect to the scramble (Silencer Select Negative Control: CTRL). Data are presented as the mean ± SD of determinations from a representative experiment performed in six independent replicates after 72 h of silencing. Asterisks indicate statistically significant differences (*p ≤0.05, **p ≤0.01, ***p ≤0.005). **Fig. S7.** Effects of BAZ1B silencing on antiestrogen-sensitive and antiestrogen-resistant breast cancer cells. Statistically significant functions highlighted by Gene Set Enrichment Analysis (GSEA) in siRNA-mediated BAZ1B kd cell transcriptome of wt (MCF7: **A**), tamoxifen (MCF7 TAM-R; **B**)- or fulvestrant/ICI (MCF7 ICI-R; **C**)-resistant BC cells. NES: Negative Normalized Enrichment Score, FDR: False Discovery Rate. **Fig. S8.** Analysis of the influence of BAZ1B, Dot1L and menin co-expression in luminal-like BC clinical outcome. Kaplan-Meier curves, generated using the Kaplan-Meier Plotter, showing the probability of overall (**A**; 205 low and 549 high samples, respectively) and relapse-free (**B**: 641 low and 261 high samples, respectively) survival of ERα+ BC patients according to BAZ1B, Dot1L and menin concomitant mRNA expression levels.

**Additional file 2: Table S1.** Differentially expressed transcripts in common between EPZ- and MI-2-treated MCF-7 cells.

**Additional file 3:** **Table S2.** Masa Spectrometry data.

**Additional file 4:** **Table S3.** Common Dot1L and Menin nuclear interactors.

**Additional file 5:** **Table S4.** Complex analysis of Dot1L and Menin common nuclear interactomes.

**Additional file 6:** Uncropped blots from images in the manuscript.

## Data Availability

All the data that support the findings of this study are available from the corresponding author upon reasonable request. The MS proteomics data related to this article have been deposited to the ProteomeXchange Consortium via the PRIDE partner repository [65] with the data set identifier PXD026864 for interaction proteomics data sets comprising CTRLs, Dot1L and menin1 samples from MCF-7 cell lines. The Raw RNA sequencing data related to CTRL and BAZ1B siRNA in AE-sensitive and AE-resistant MCF-7 cells have been deposited in the EBI ArrayExpress database (http://www.ebi.ac.uk/arrayexpress) with accession number E-MTAB-11142. Uncropped blots from images in the manuscript are included in Additional file 6.

## Abbreviations

BAZ1B: Bromodomain adjacent to zinc finger domain 1B
BC: Breast cancer
CPTAC: Clinical proteomic tumor analysis consortium
ChIP: Chromatin immunoprecipitation
Dot1L: Histone lysine N-methyltransferase, H3 lysine-79 specific
ERα: Estrogen receptor alpha
GSEA: Gene set enrichment analysis
ICI: Fulvestrant
KD: Knockdown
MEN1: Multiple endocrine neoplasia type 1 gene
MLL: Mixed-lineage leukemia
MS: Mass spectrometry
TAM: 4-Hydroxytamoxifen
TCGA: The cancer genome atlas
