# Supplementary material for: Combinatorial targeting of a chromatin complex comprising Dot1L, menin and the tyrosine kinase BAZ1B reveals a new therapeutic vulnerability of endocrine therapy-resistant breast cancer
Source: Breast Cancer Res. 2022 Jul 18;24:52. doi: 10.1186/s13058-022-01547-7 (PMC9290241; doi:10.1186/s13058-022-01547-7)
Supplement: Supplementary file 1 — Additional file 1: Fig. S1. Dot1L and menin expression in breast cancer. Dot1L (A, left panel) and menin (A, right panel) mRNA levels in normal and luminal breast cancer samples from The Cancer Genome Atlas (TCGA), analyzed with UALCAN. B) Dot1L and menin mRNA co-expression from two additional BC patient datasets from TCGA. Only expression values above the first quartile were considered. Fig. S2. Dot1L, menin and ERα binding to MCF-7 cell chromatin. Heatmap (left panel) showing read density within 10 kb regions centred on Dot1L, menin and ERα binding sites in MCF-7 cells. Control (CTRL) was obtained using nonspecific Abs. Overrepresented transcription factor binding motifs within Dot1L+menin+ERα binding sites (green) or Dot1L+menin binding sites (blue) are reported in the word cloud (right panel). Fig. S3. Impact of Dot1L and menin blockade on gene expression and cell proliferation in BC cells. Bar charts from KEGG functional enrichment analysis showingstatistically significant pathways deregulated upon MCF-7 cell treatment with EPZ (A) or MI-2 (B). Combenefit software was used to generate dose-response surface curves in BT474 (C), T47D (D) and Zr75.1 (E) BC cells according to D–R Lowe model, combining increasing dose of EPZ and MI-136 for 9 days. Color scale bar indicate the level of synergy (blue) or antagonism (red) at each combination. Effect of siRNA-mediated Dot1L and menin silencing on AE-sensitive (MCF7; F) and tamoxifen (MCF7 TAM-R; G)- or fulvestrant/ICI (MCF7 ICIR; H)-resistant BC cells assessed by RT-qPCR (top) and western blot (middle) assays. RT-qPCR results are shown as mean ± SD of triplicate determinations from a representative experiment after 72h of silencing. Western blot show Dot1L or menin protein levels following their silencing. β-actin (ACTB) was used as control. Effect of siRNA-mediated Dot1L and menin silencing on AE-sensitive (MCF7; I) and tamoxifen (MCF7 TAM-R; J)- or fulvestrant/ICI (MCF7 ICIR; J)-resistant BC cells assessed on cell pro [file 13058_2022_1547_MOESM1_ESM.docx]

**SUPPLEMENTARY INFORMATION**

**Combinatorial targeting of a chromatin complex comprising Dot1L, menin and the tyrosine kinase BAZ1B reveals a new therapeutic vulnerability of endocrine therapy-resistant breast cancer**

Annamaria Salvati *et al.*

**SUPPLEMENTARY FIGURES**

**Supplementary Figure S1**

**
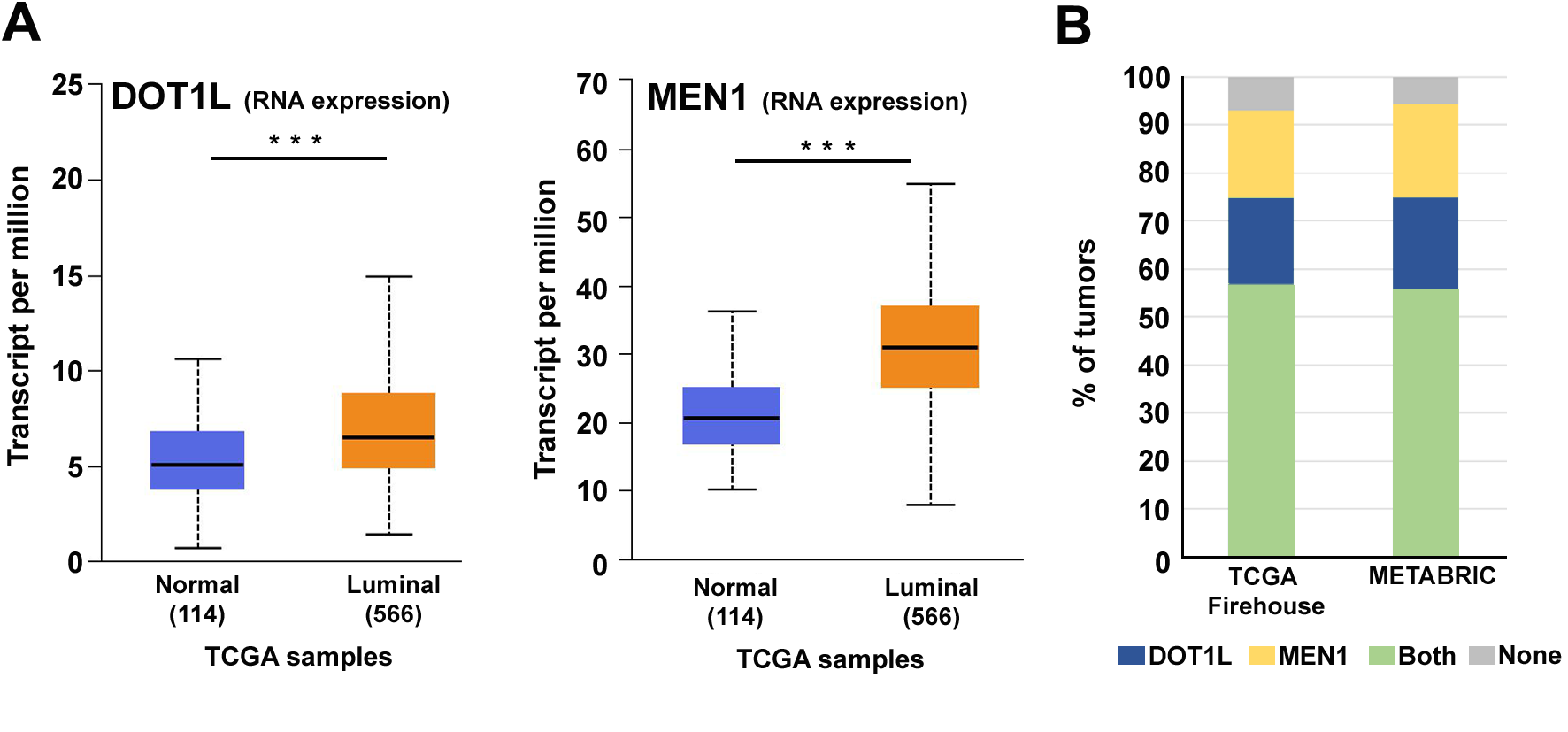
**

**Supplementary Fig. S1. Dot1L and menin expression in breast cancer**. Dot1L (**A**, left panel) and menin (**A**, right panel) mRNA levels in normal and luminal breast cancer samples from The Cancer Genome Atlas (TCGA), analysed with UALCAN. **B)** Dot1L and menin mRNA co-expression from two additional BC patient datasets from TCGA. Only expression values above the first quartile were considered.

**Supplementary Figure S2**

**
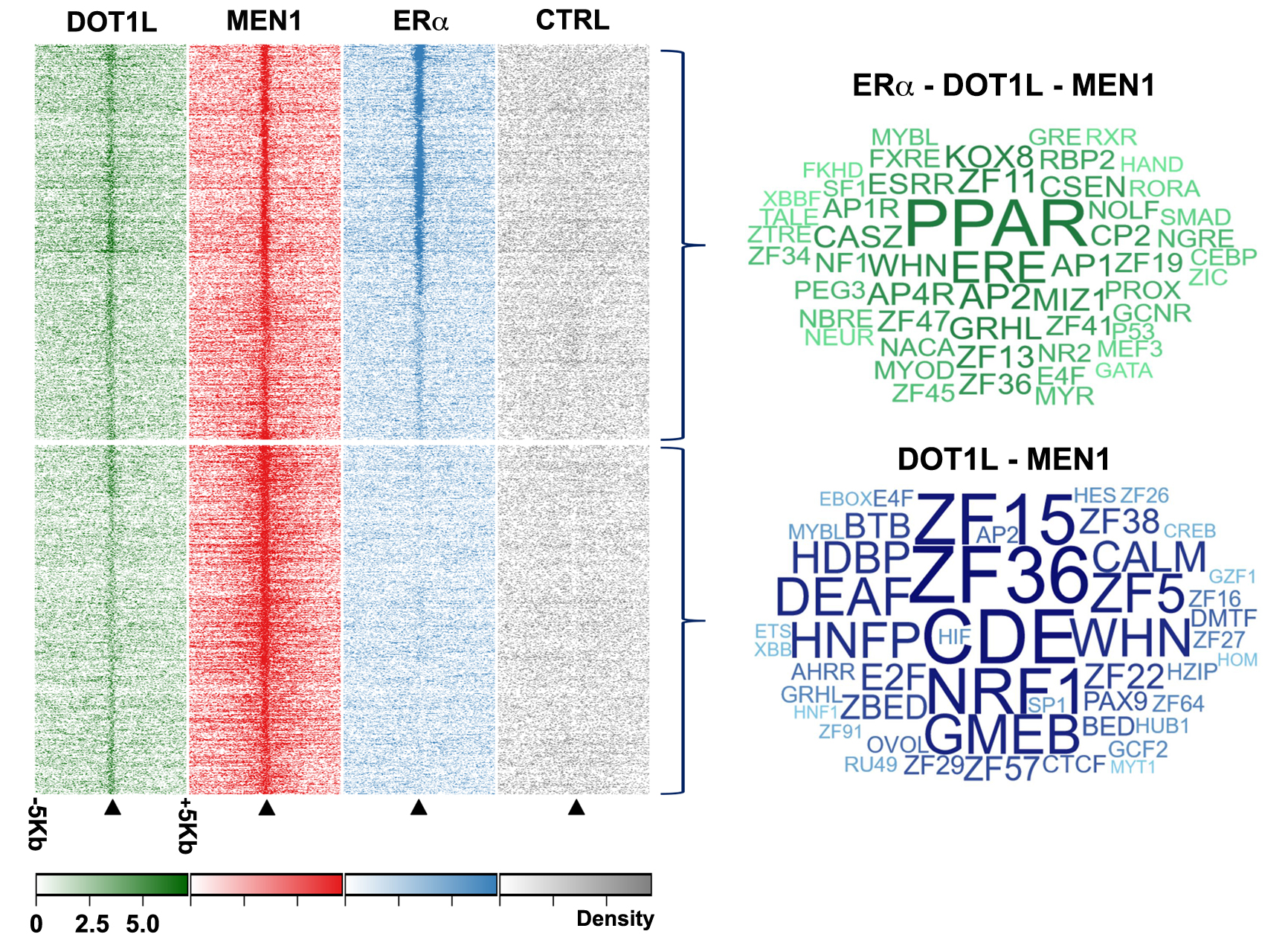
**

**Supplementary Fig. S2. Dot1L, menin and ER**α **binding to MCF-7 cell chromatin.** Heatmap (left panel) showing read density within 10-kb regions centred on Dot1L, menin and ERα binding sites in MCF-7 cells. Control (CTRL) was obtained using nonspecific Abs. Over-represented transcription factor binding motifs within Dot1L+menin+ERα binding sites (green) or Dot1L+menin binding sites (blue) are reported in the word cloud (right panel).

**Supplementary Figure S3**

**
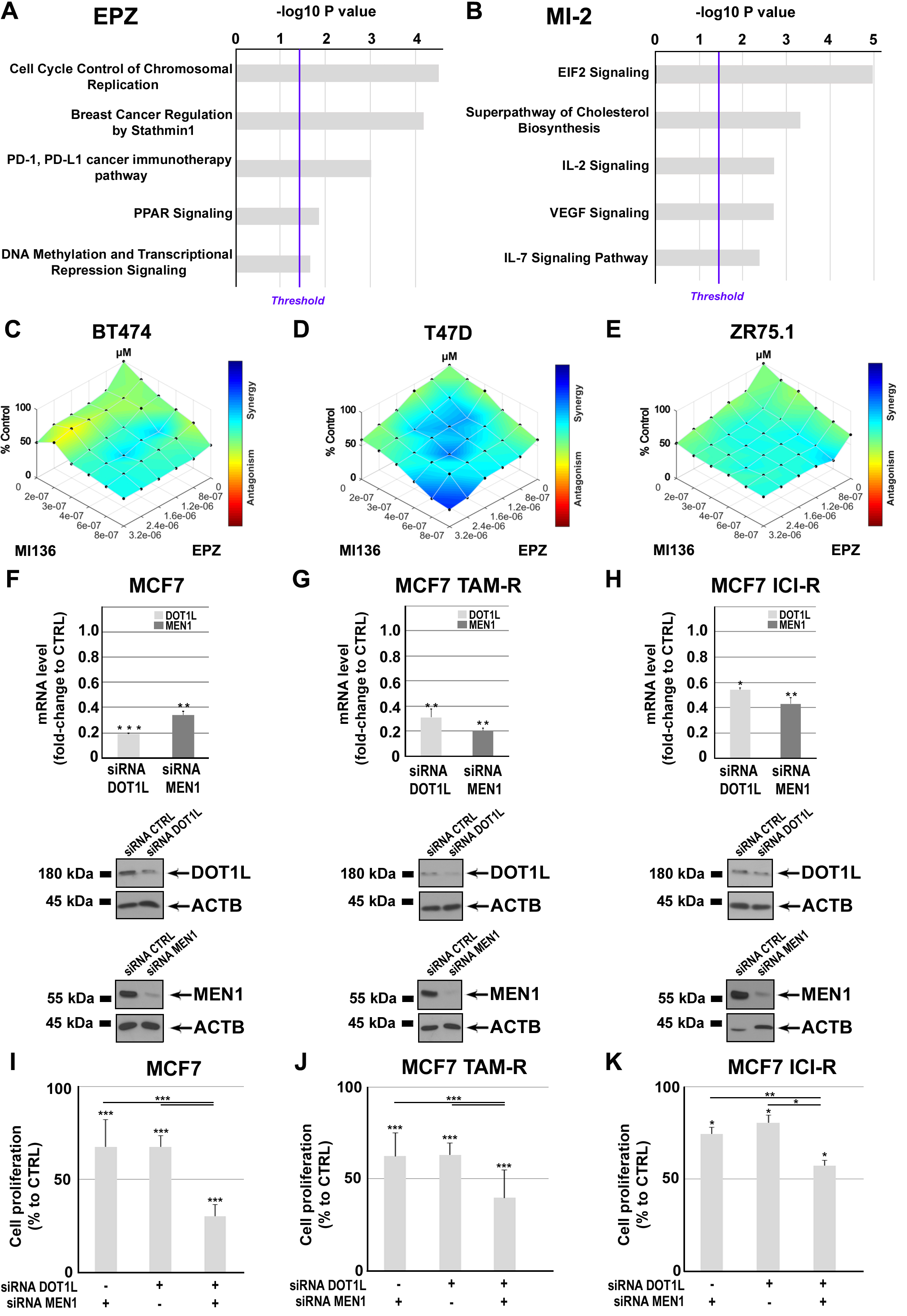
**

**Supplementary Fig. S3. Impact of Dot1L and menin blockade on gene expression and cell proliferation in BC cells.** Bar charts from KEGG functional enrichment analysis showingstatistically significant pathways deregulated upon MCF-7 cell treatment with EPZ (**A**) or MI-2 (**B**). Combenefit software was used to generate dose-response surface curves in BT474 (**C**), T47D (**D**) and Zr75.1 (**E**) BC cells according to D-R Lowe model, combining increasing dose of EPZ and MI-136 for 9 days. Color scale bar indicate the level of synergy (blue) or antagonism (red) at each combination. Effect of siRNA-mediated Dot1L and menin silencing on AE-sensitive (MCF7; **F**) and tamoxifen- (MCF7 TAM-R; **G**) or Fulvestrant/ICI- (MCF7 ICIR; **H**) resistant BC cells assessed by RT-qPCR (top) and western blot (middle) assays. RT-qPCR results are shown as mean ± SD of triplicate determinations from a representative experiment after 72h of silencing. Western blot show Dot1L or menin protein levels following their silencing. β-actin (ACTB) was used as control. Effect of siRNA-mediated Dot1L and menin silencing on AE-sensitive (MCF7; **I**) and tamoxifen- (MCF7 TAM-R; **J**) or Fulvestrant/ICI- (MCF7 ICIR; **J**) resistant BC cells assessed on cell proliferation. MTT data are presented as the mean ±SD from six independent replicates after 72h of silencing. Asterisks indicate statistically significant differences (*p ≤0.05, **p ≤0.01, ***p ≤0.005) to CTRL or to single treatment (black bars).

**Supplementary Figure S4**

**
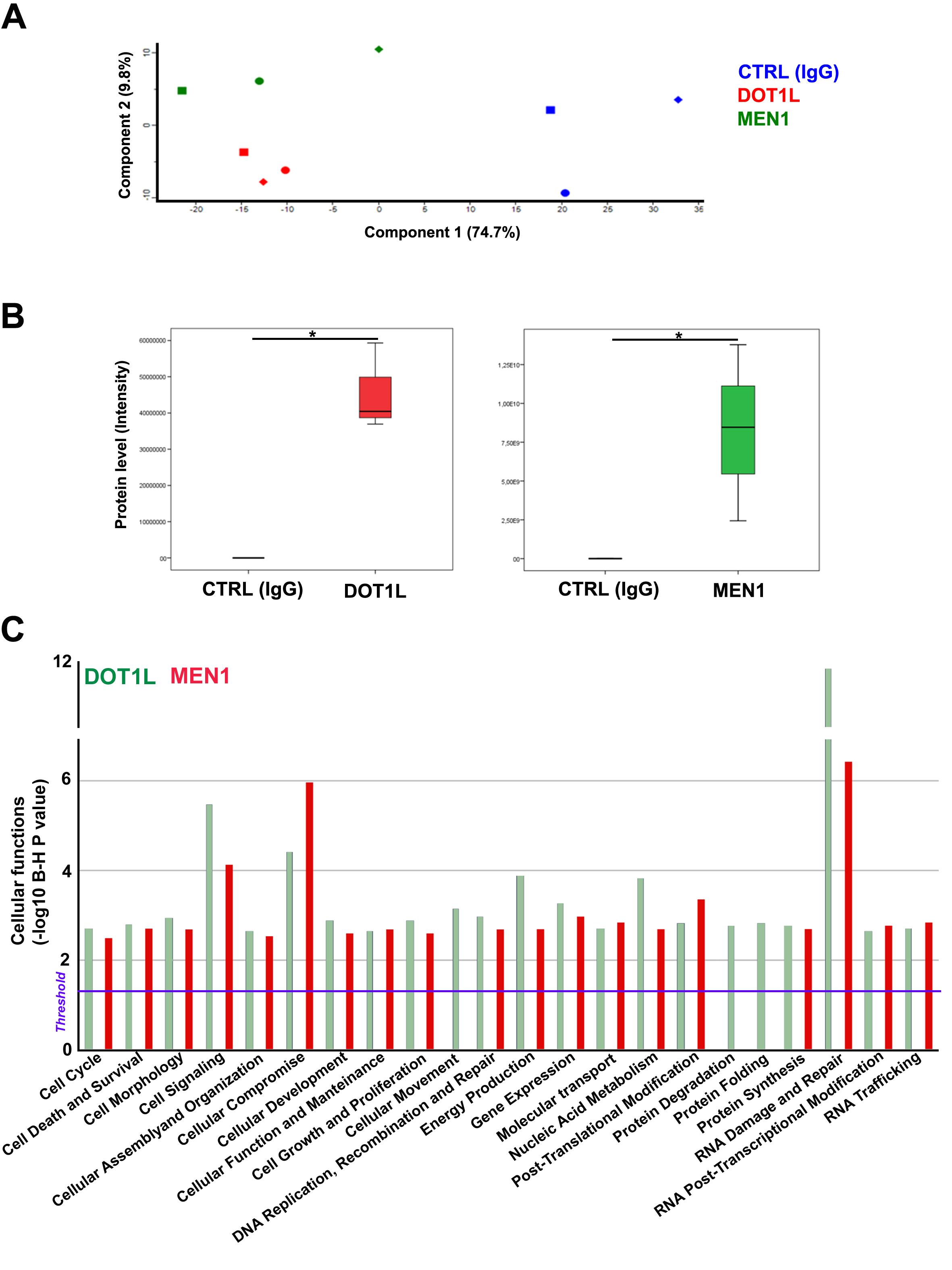
**

**Supplementary Fig. S4. Technical and functional evaluation of Dot1L and menin interactome analysis by mass spectrometry. A)** Principal Component Analysis (PCA) of the mass spectrometry (MS) results obtained from three biological replicates of control (CTRL; IgG: blue), Dot1L (red) and menin (MEN1; green) samples. Numbers between parentheses indicate percentage of total variance. **B)** Box plot showing Dot1L (red) and menin (green) protein levels detected in CTRLs and samples by MS. Asterisks indicate statistically significant differences (**p* ≤0.01). **C)** Results of IPA (Ingenuity Pathway Analysis) functional analysis showing statistically significant molecular functions enriched in Dot1L (green) and menin (red) interactomes.

**Supplementary Figure S5**

**
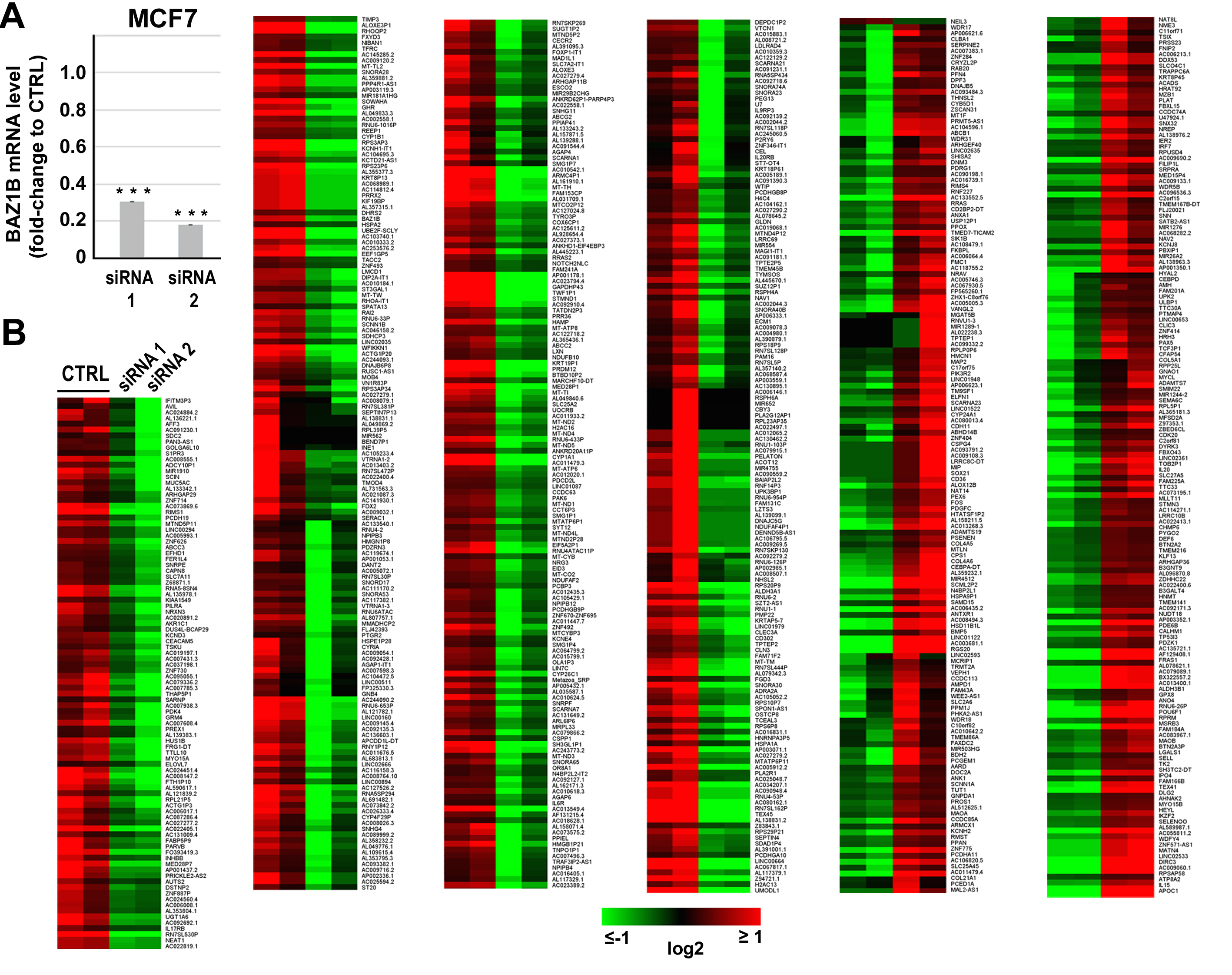
**

**Supplementary Fig. S5 Impact of BAZ1B silencing on gene expression in MCF-7 BC cells. A)** RT-qPCR analysis of BAZ1B mRNA level after silencing with two different siRNAs compared to scramble (Silencer Select Negative Control: CTRL). RT-qPCR results shown are the mean ± SD of multiple determinations from a representative experiment. Asterisks indicate statistically significant differences (***p ≤0.005). **B)** Heatmap showing top down- (green) and up (red) -regulated transcripts, fold-change ≤ and ≥ |1.5|, following BAZ1B silencing with both siRNAs, compared to scramble (Silencer Select Negative Control: CTRL)

**
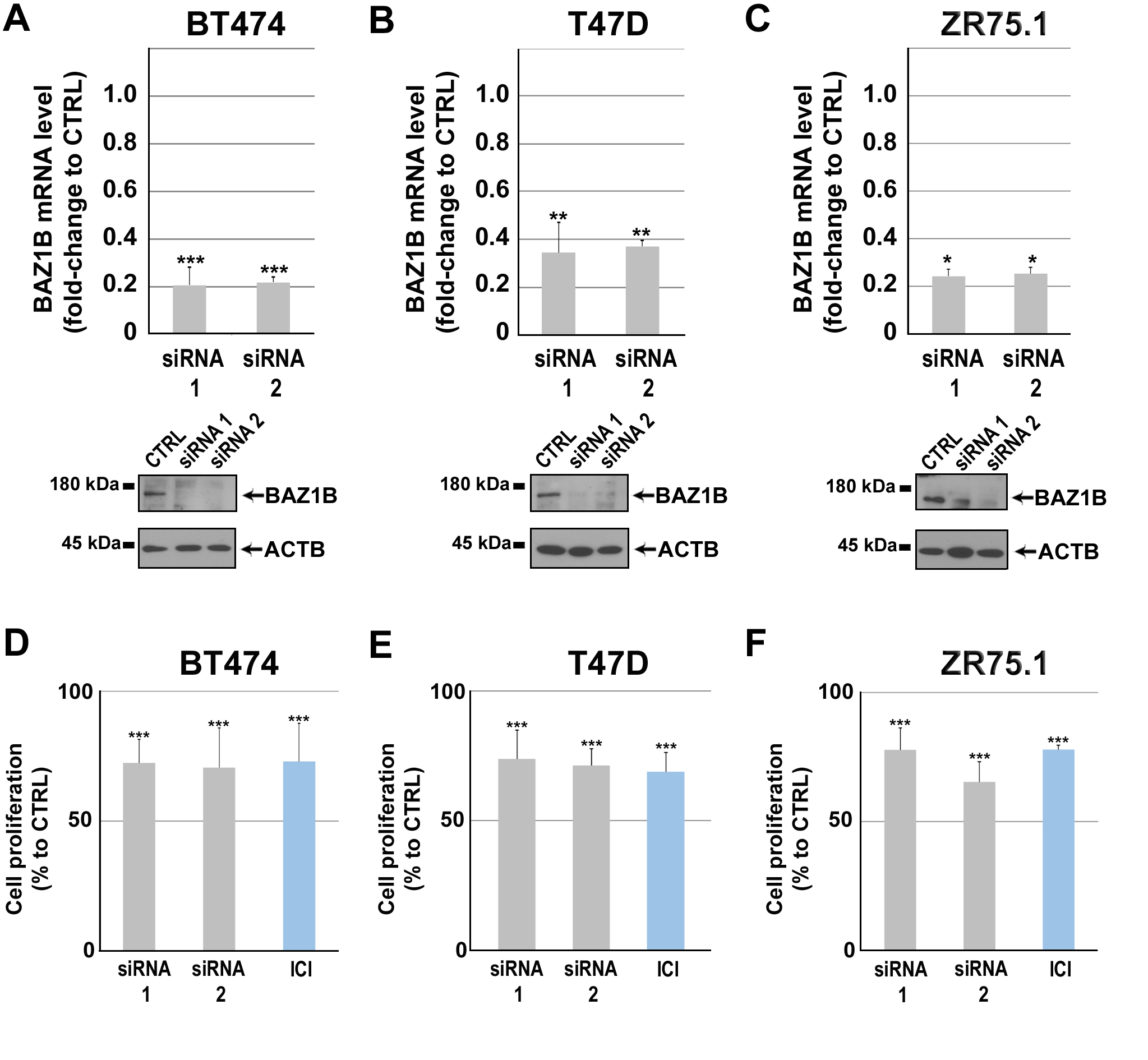
Supplementary Figure S6**

Supplementary Fig. S6. **Effect of BAZ1B silencing in luminal-like BC cell models**. RT-qPCR (upper panel) and western blot analysis of BAZ1B mRNA and protein levels following BAZ1B silencing in BT474 **(A)**, T47D **(B)** and ZR75.1 **(C)** BC cells. RT-qPCR results are shown as mean ± SD of triplicate determinations from a representative experiment after 72h of silencing. Western blot shows BAZ1B protein level following BAZ1B silencing. β-actin (ACTB) was used as control Cell proliferation rate performed following BAZ1B silencing or treatment with fulvestrant (ICI; 100 nM) in BT474 (**D**), T47D (**E**) and ZR75.1 (**F**) BC cells. All data are analyzed respect to the scramble (Silencer Select Negative Control: CTRL). Data are presented as the mean ± SD of determinations from a representative experiment performed in six independent replicates after 72h of silencing. Asterisks indicate statistically significant differences (*p ≤0.05, **p ≤0.01, ***p ≤0.005).

**Supplementary Figure S7**

**
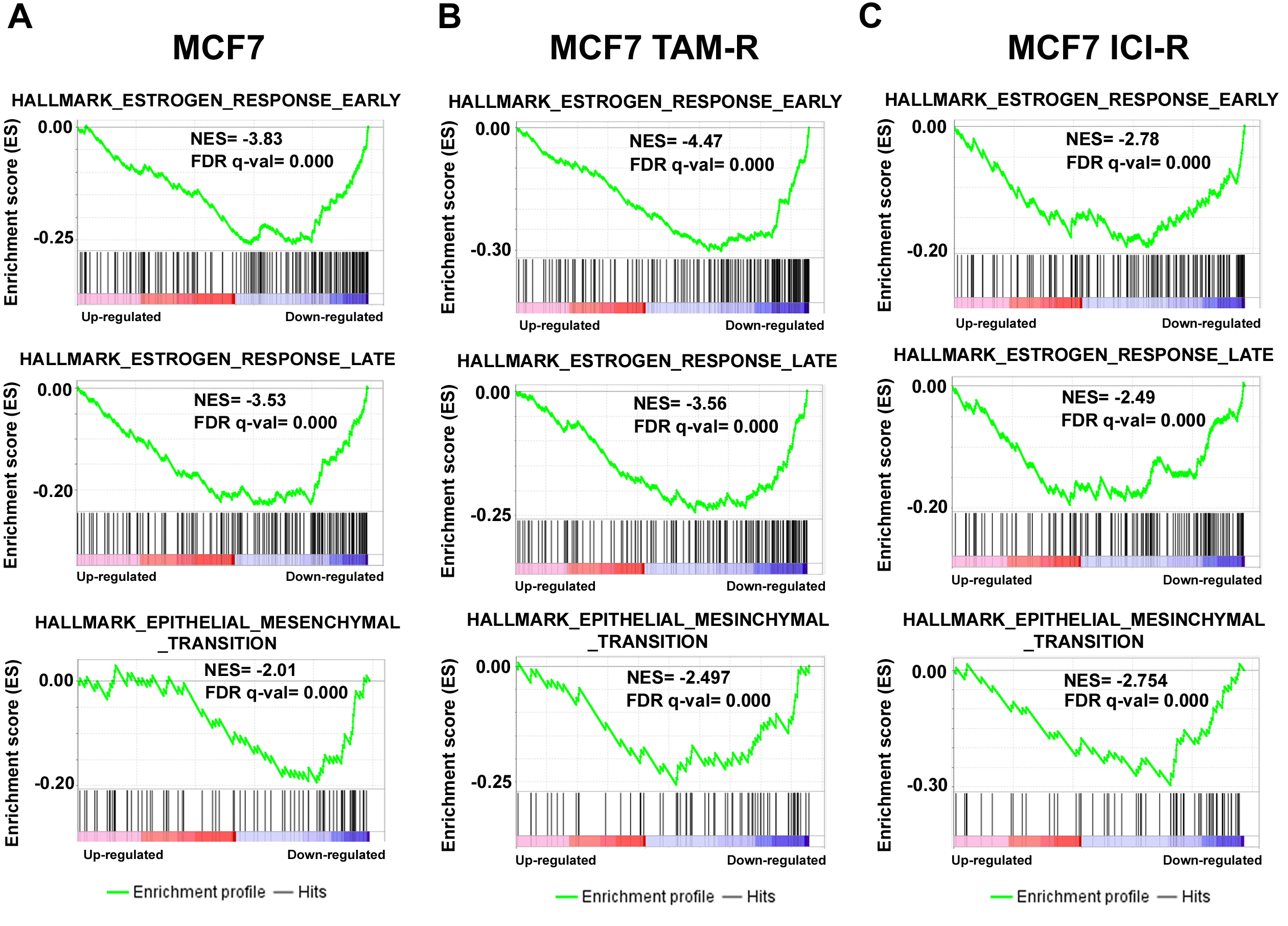
**

**Supplementary Fig. S6. Effects of BAZ1B silencing on antiestrogen-sensitive and resistant breast cancer cells.** Statistically significant functions highlighted by Gene Set Enrichment Analysis (GSEA) in siRNA-mediated BAZ1B kd cell transcriptome of *wt* (MCF7: **A**), tamoxifen- (MCF7 TAM-R; **B)** or fulvestrant**/**ICI**-** (MCF7 ICI-R; **C)** resistant BC cells. NES: Negative Normalized Enrichment Score, FDR: False Discovery Rate.

**Supplementary Figure S8**

**
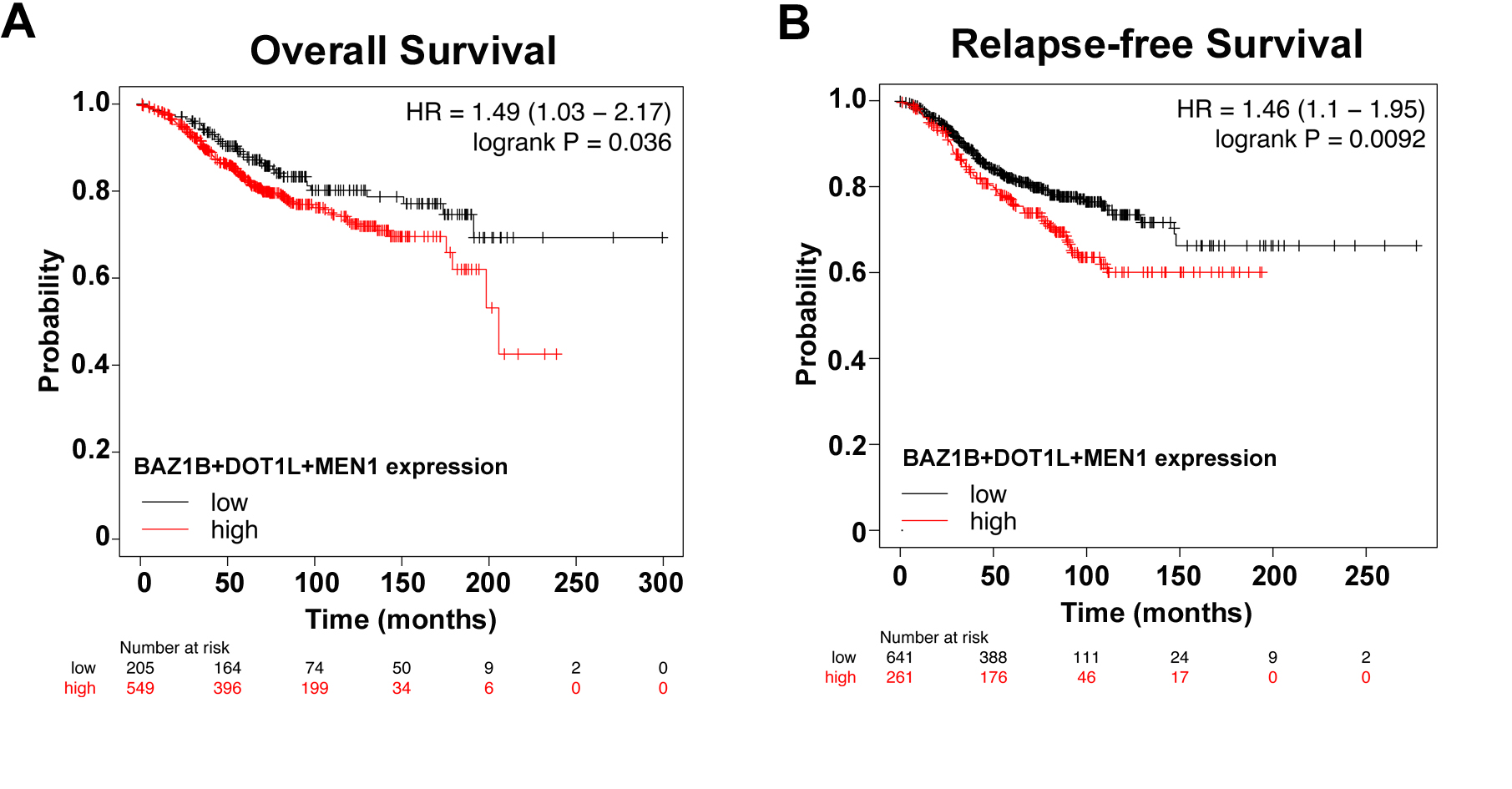
**

**Supplementary Fig. S8. Analysis of the influence of BAZ1B, Dot1L and menin co-expression in luminal-like BC clinical outcome.** Kaplan-Meier curves, generated using the Kaplan-Meier Plotter, showing the probability of overall (A; 205 low and 549 high samples, respectively) and relapse-free (B: 641 low and 261 high samples, respectively) survival of ERα+ BC patients according to BAZ1B, Dot1L and menin concomitant mRNA expression levels.
