# Supplementary material for: Combinatorial targeting of a chromatin complex comprising Dot1L, menin and the tyrosine kinase BAZ1B reveals a new therapeutic vulnerability of endocrine therapy-resistant breast cancer
Source: Breast Cancer Res. 2022 Jul 18;24:52. doi: 10.1186/s13058-022-01547-7 (PMC9290241; doi:10.1186/s13058-022-01547-7)

Uncropped blots Fig. 1A

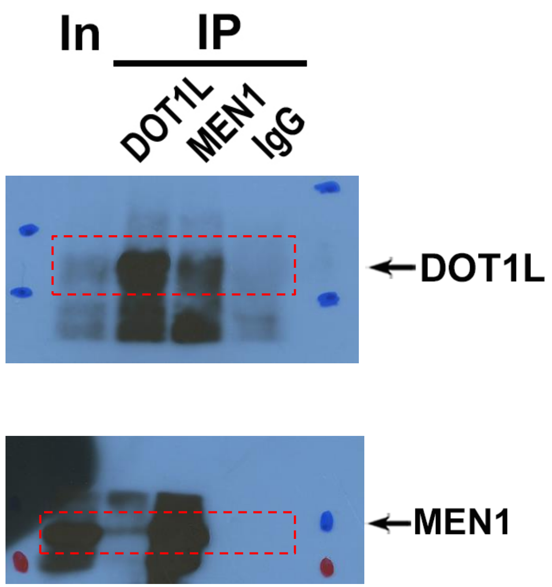

Uncropped blots Fig. 3A

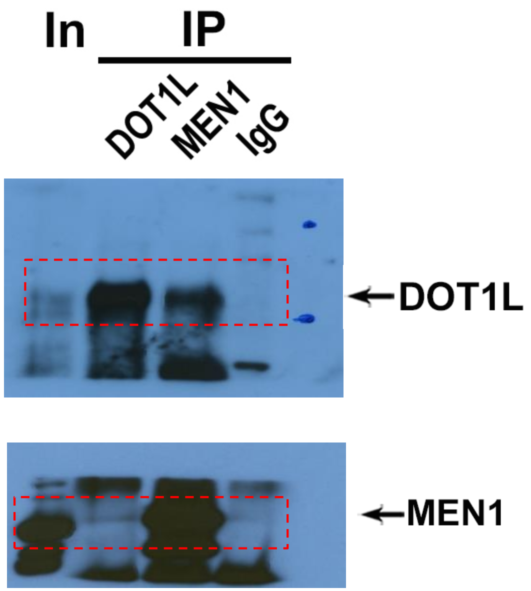

Uncropped blots Fig. 4B

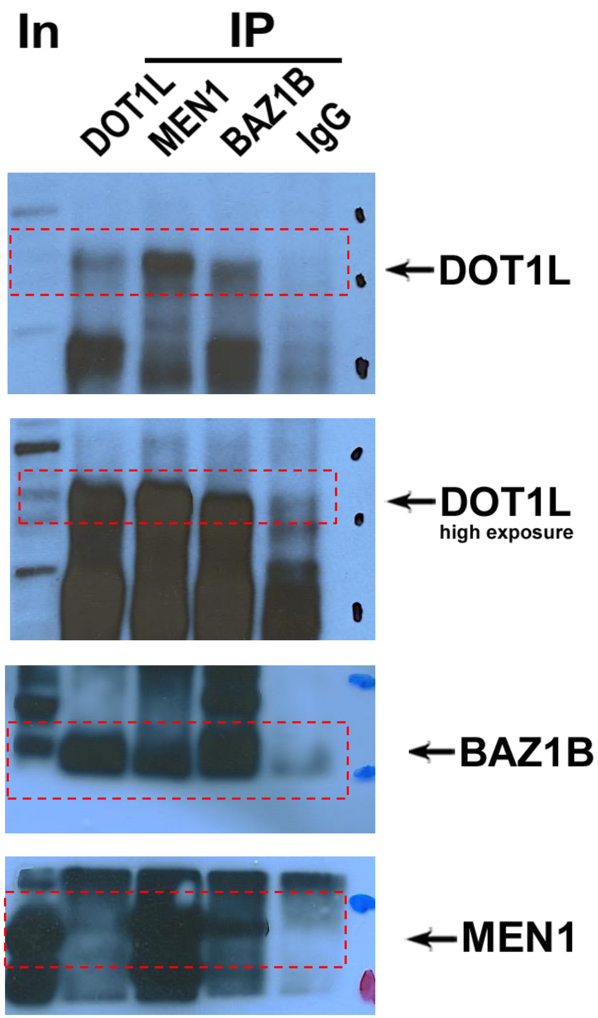

Uncropped blots Fig. 6D

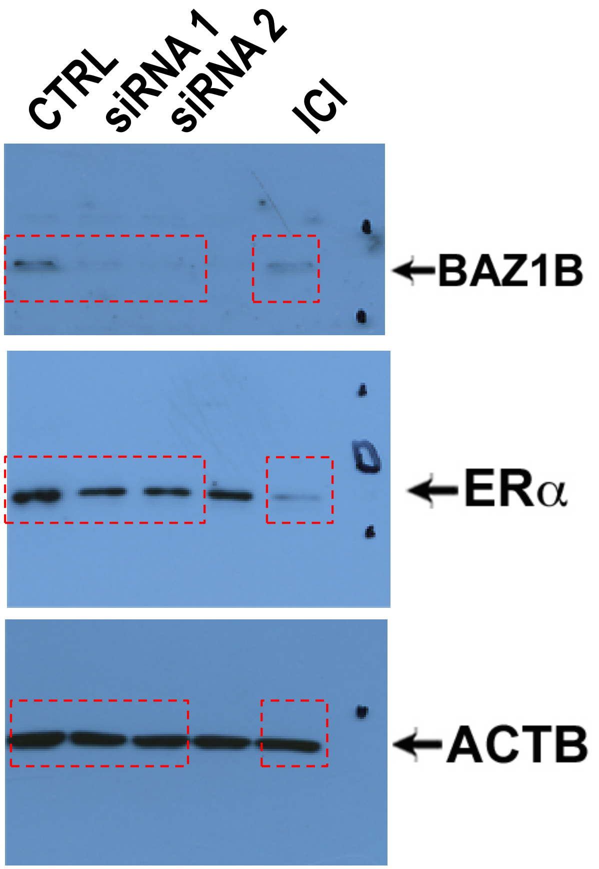

Uncropped blots Fig. 6E

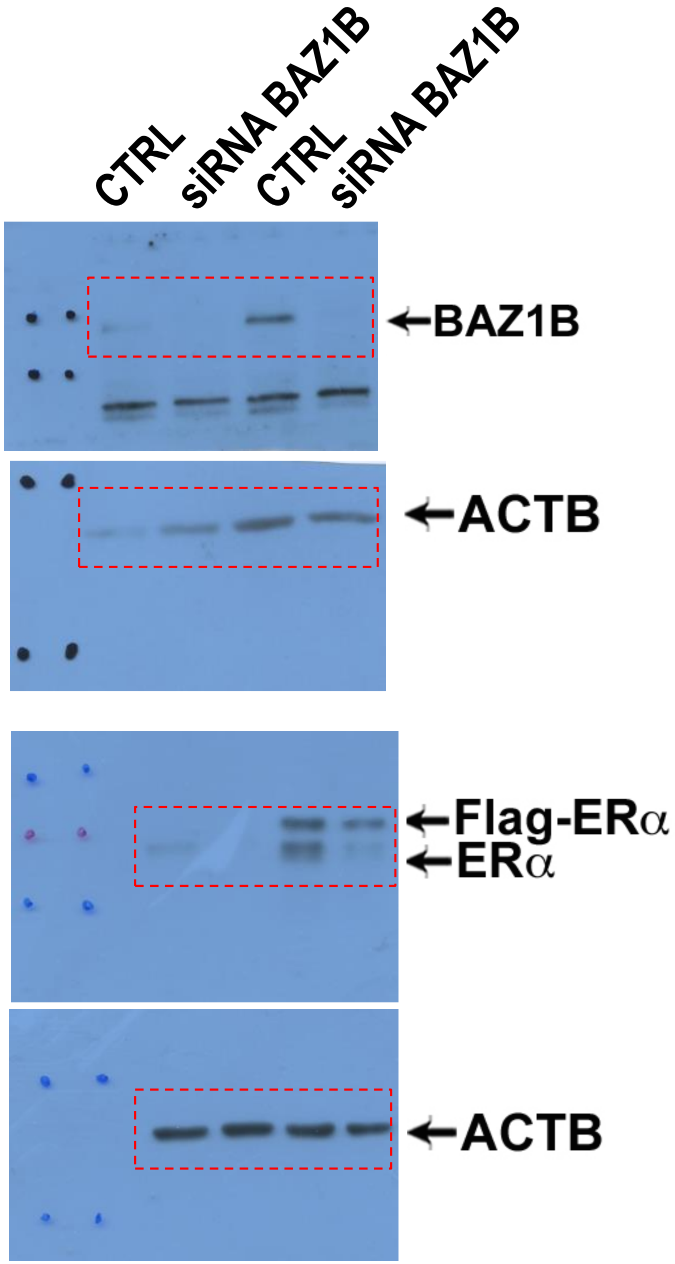

Uncropped blots Fig. 7F

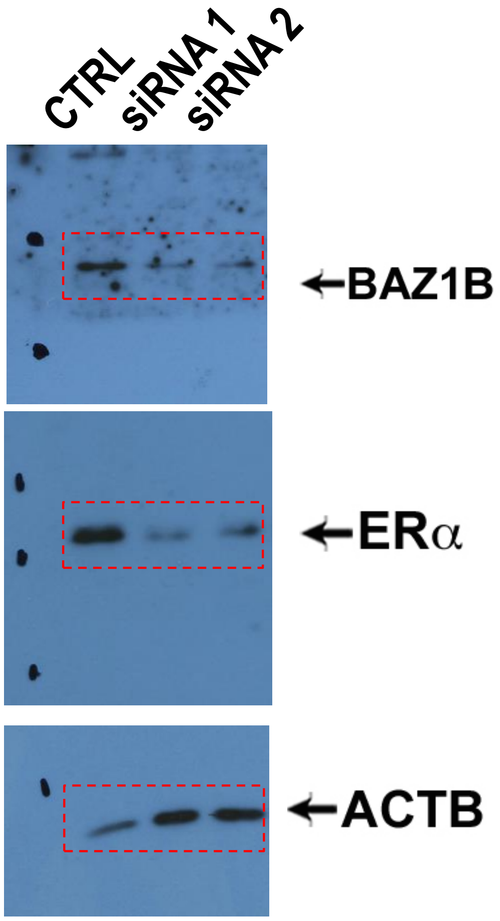

Uncropped blots Fig. 7I

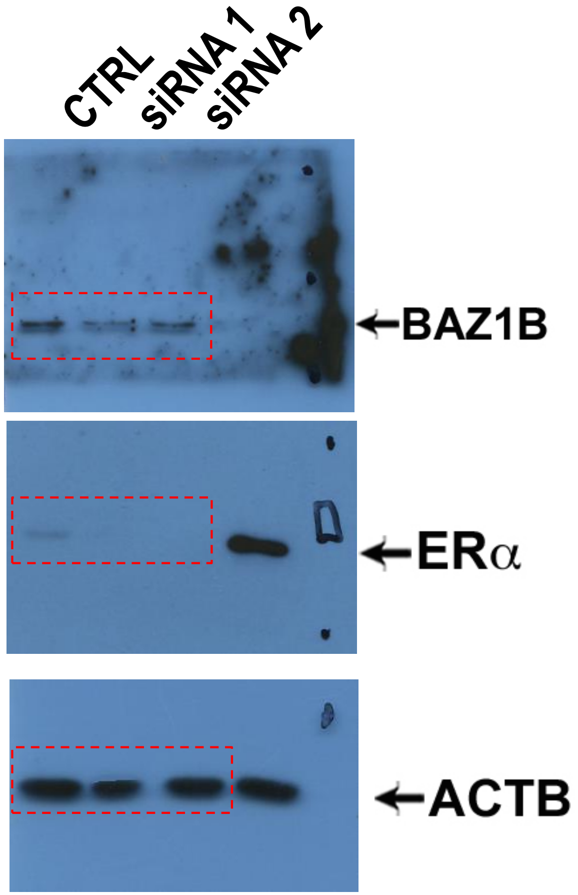

Uncropped blots Fig. S3 (DOT1L)

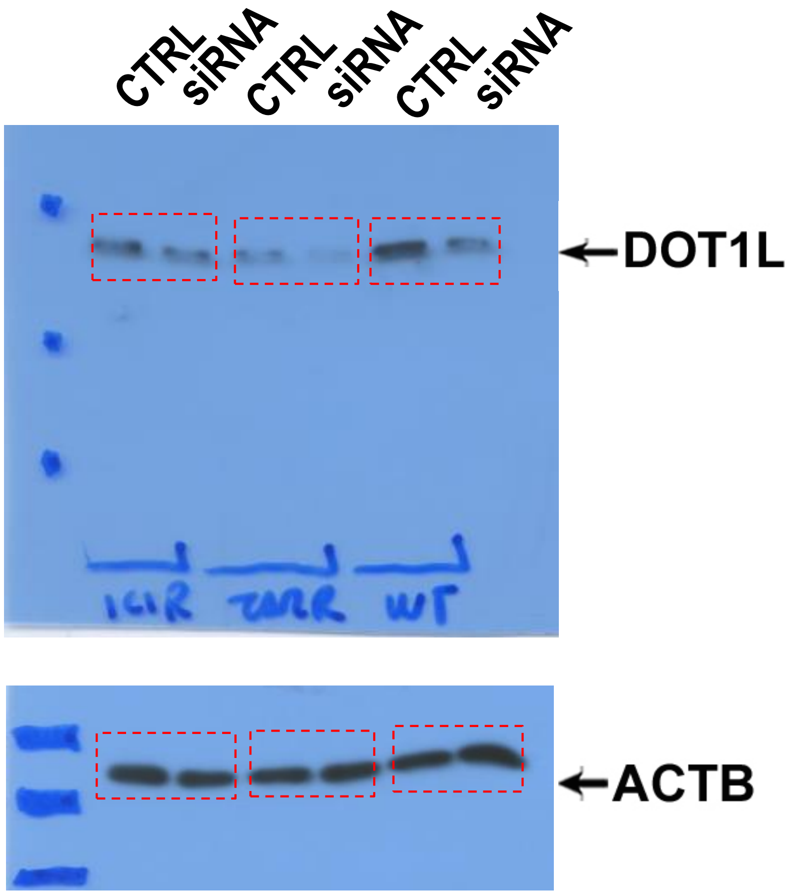

Uncropped blots Fig. S3 (MEN1)

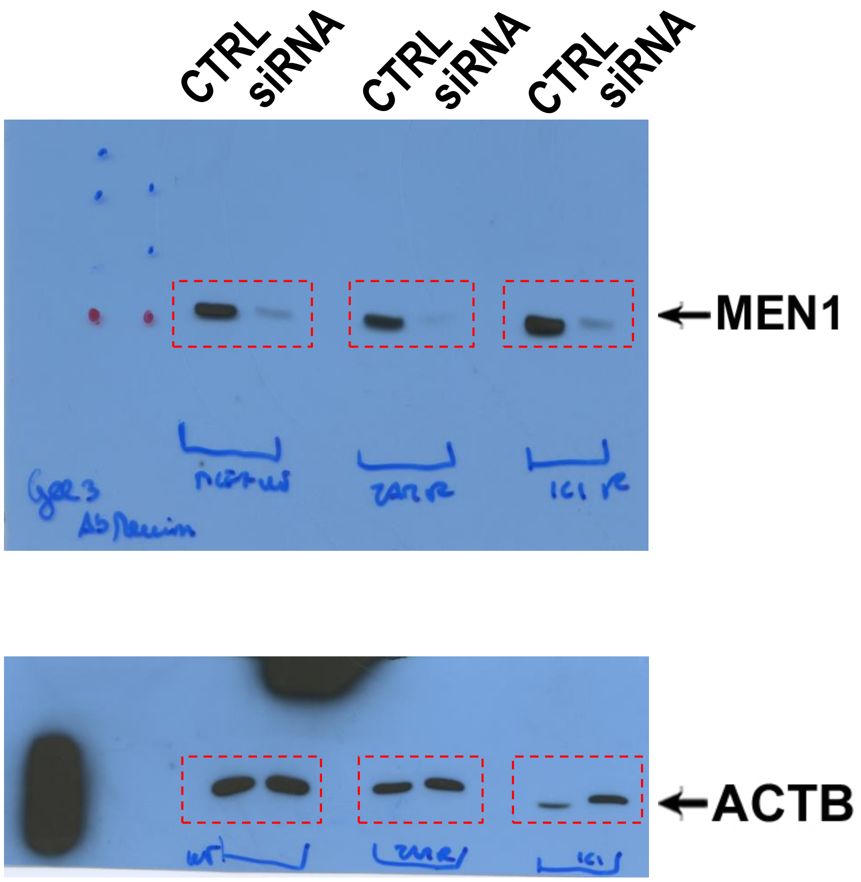

Uncropped blots Fig. S6A-C

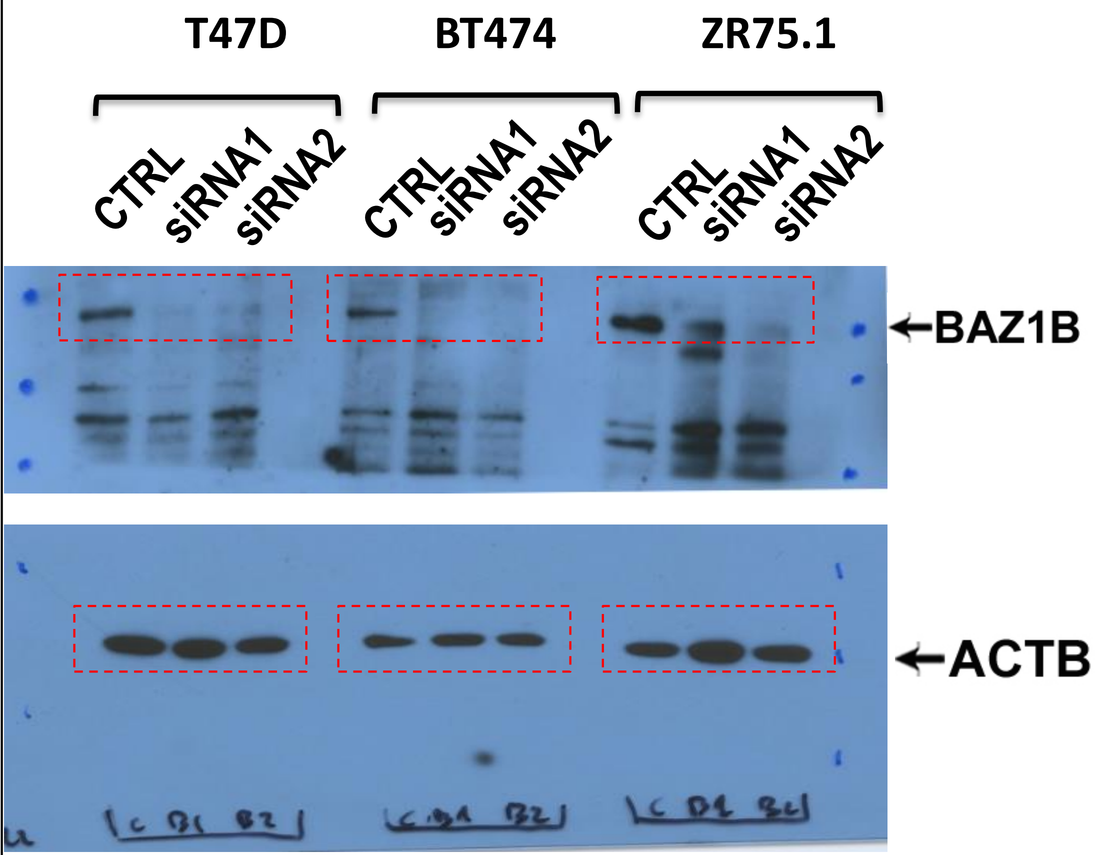

Supplement: Supplementary file 6 — Additional file 6: Uncropped blots from images in the manuscript. [file 13058_2022_1547_MOESM6_ESM.pdf]
